# Solid-solution-induced lattice hardening reinforces perovskite stiffness for stable solar cells

**DOI:** 10.1093/nsr/nwag484

**Published:** 2026-08-25

**Authors:** Jiahao Liang, Hailin Li, Tian Chen, Shengcai Zhu, Hepeng Wang, Zhouti Wang, Yuecheng Hu, Huahui Chen, Wenchang Huo, Ming Fang, Wenjun Liu, Hao Lin, Jiancan Yu, Jiangsheng Xie, Pingqi Gao

**Affiliations:** School of Materials, Shenzhen Campus of Sun Yat-sen University, Shenzhen 518107, China; Institute for Solar Energy Systems, Guangdong Engineering Technology Research Center for Sustainable Photovoltaic Technology and Equipment, State Key Laboratory of Optoelectronic Materials and Technologies, Sun Yat-sen University, Guangzhou 510275, China; College of Materials Science and Engineering, Shenzhen University, Shenzhen 518060, China; School of Materials, Shenzhen Campus of Sun Yat-sen University, Shenzhen 518107, China; Institute for Solar Energy Systems, Guangdong Engineering Technology Research Center for Sustainable Photovoltaic Technology and Equipment, State Key Laboratory of Optoelectronic Materials and Technologies, Sun Yat-sen University, Guangzhou 510275, China; School of Materials, Shenzhen Campus of Sun Yat-sen University, Shenzhen 518107, China; Institute for Solar Energy Systems, Guangdong Engineering Technology Research Center for Sustainable Photovoltaic Technology and Equipment, State Key Laboratory of Optoelectronic Materials and Technologies, Sun Yat-sen University, Guangzhou 510275, China; School of Materials, Shenzhen Campus of Sun Yat-sen University, Shenzhen 518107, China; School of Materials, Shenzhen Campus of Sun Yat-sen University, Shenzhen 518107, China; Institute for Solar Energy Systems, Guangdong Engineering Technology Research Center for Sustainable Photovoltaic Technology and Equipment, State Key Laboratory of Optoelectronic Materials and Technologies, Sun Yat-sen University, Guangzhou 510275, China; School of Materials, Shenzhen Campus of Sun Yat-sen University, Shenzhen 518107, China; Institute for Solar Energy Systems, Guangdong Engineering Technology Research Center for Sustainable Photovoltaic Technology and Equipment, State Key Laboratory of Optoelectronic Materials and Technologies, Sun Yat-sen University, Guangzhou 510275, China; School of Materials, Shenzhen Campus of Sun Yat-sen University, Shenzhen 518107, China; Institute for Solar Energy Systems, Guangdong Engineering Technology Research Center for Sustainable Photovoltaic Technology and Equipment, State Key Laboratory of Optoelectronic Materials and Technologies, Sun Yat-sen University, Guangzhou 510275, China; School of Materials, Shenzhen Campus of Sun Yat-sen University, Shenzhen 518107, China; Institute for Solar Energy Systems, Guangdong Engineering Technology Research Center for Sustainable Photovoltaic Technology and Equipment, State Key Laboratory of Optoelectronic Materials and Technologies, Sun Yat-sen University, Guangzhou 510275, China; School of Materials, Shenzhen Campus of Sun Yat-sen University, Shenzhen 518107, China; Institute for Solar Energy Systems, Guangdong Engineering Technology Research Center for Sustainable Photovoltaic Technology and Equipment, State Key Laboratory of Optoelectronic Materials and Technologies, Sun Yat-sen University, Guangzhou 510275, China; College of Materials Science and Engineering, Shenzhen University, Shenzhen 518060, China; College of Materials Science and Engineering, Shenzhen University, Shenzhen 518060, China; School of Materials, Shenzhen Campus of Sun Yat-sen University, Shenzhen 518107, China; Institute for Solar Energy Systems, Guangdong Engineering Technology Research Center for Sustainable Photovoltaic Technology and Equipment, State Key Laboratory of Optoelectronic Materials and Technologies, Sun Yat-sen University, Guangzhou 510275, China; School of Materials, Shenzhen Campus of Sun Yat-sen University, Shenzhen 518107, China; Institute for Solar Energy Systems, Guangdong Engineering Technology Research Center for Sustainable Photovoltaic Technology and Equipment, State Key Laboratory of Optoelectronic Materials and Technologies, Sun Yat-sen University, Guangzhou 510275, China; School of Materials, Shenzhen Campus of Sun Yat-sen University, Shenzhen 518107, China; Institute for Solar Energy Systems, Guangdong Engineering Technology Research Center for Sustainable Photovoltaic Technology and Equipment, State Key Laboratory of Optoelectronic Materials and Technologies, Sun Yat-sen University, Guangzhou 510275, China; School of Materials, Shenzhen Campus of Sun Yat-sen University, Shenzhen 518107, China; Institute for Solar Energy Systems, Guangdong Engineering Technology Research Center for Sustainable Photovoltaic Technology and Equipment, State Key Laboratory of Optoelectronic Materials and Technologies, Sun Yat-sen University, Guangzhou 510275, China; Sichuan Yat-sen Innovation Center of Photovoltaic Industry, Yibin 644609, China

**Keywords:** perovskite solar cells, lattice hardening, Young’s modulus, thermal expansion, hole transport layer

## Abstract

The operational instability of perovskite photovoltaics is mainly attributed to the soft, ionic nature of its lattice. However, the quantification and intrinsic tuning strategy of perovskite lattice stiffness, and its correlation with device performance and stability have not been well explored. Here, we propose a solid-solution-induced lattice hardening strategy to enhance the stiffness of perovskite through partial substitution of lead ions with lanthanum ions. The reinforcement mechanism stems from the beneficial lattice contraction and bond-strengthening induced by the lanthanum dopant with smaller ionic radius and stronger chemical interaction, which collectively yield a denser and more rigid framework. Consequently, the strategy obviously enhances the perovskite stiffness with Young’s modulus increasing from 27.96 to 35.71 GPa. The enhanced lattice stiffness reduces the thermal expansion coefficient, suppresses defect formation and ion migration, thereby mitigating non-radiative recombination and structural degradation under external stress. By employing 4-isopropyl-4′-methyldiphenyliodonium tetrakis(pentafluorophenyl) borate as a stable p-dopant for the hole transport layer, the resulting device achieves a champion power conversion efficiency of 26.54% and a certified steady-state efficiency of 26.03%. The optimized devices retain >95% and >90% of their initial efficiencies after 1000 h of continuous 1-sun illumination at 65°C and under damp-heat conditions, respectively.

## Teaser

A solid-solution-induced lattice-hardening strategy reinforces the inherently soft perovskite lattice, suppressing defect formation and ion migration, and enables solar cells with a certified steady-state efficiency of 26.03% and improved stability.

## INTRODUCTION

Metal halide perovskite solar cells (PSCs) have achieved certified power conversion efficiencies exceeding 27%, rivaling conventional crystalline silicon solar cells [1,2]. However, their commercialization is hindered by the inherent instability of perovskite materials, particularly under operational stressors including heat, light, and humidity [3–6]. Compared with crystalline silicon, which is composed of chemically stable atoms through strong covalent bonds, the metal halide perovskites exhibit soft ionic lattice characteristics due to weak chemical bonds [7–9]. The perovskite structure depends on the BX_6_ octahedral framework, whose instability is fundamentally linked to the weak bonding between the B-site cation and the X-site halide anion [10,11]. The fragile B–X bonds are prone to break under external stimuli, generating a high density of point defects and enabling halide ion migration [12–15]. These processes collectively drive structural degradation, including lattice distortion, phase segregation and irreversible phase transitions [16–18].

A predominant strategy to mitigate these issues has focused on strengthening the perovskite surface and grain boundaries through passivation [19–25]. For instance, pseudohalide or trifluoromethanesulfonate anions can replace the unstable halides, forming stronger bonds with uncoordinated Pb^2+^ ions and thereby passivating defects and stabilizing the surface structure [26,27]. In addition, other lead oxysalt layers can also afford much better resistance to detrimental stimuli through forming strong chemical bonds with the perovskite surface [18,28,29]. While these passivation strategies have improved efficiency and stability by suppressing external degradation triggers, their effect is inherently localized to interfaces and does not address the intrinsic softness and dynamic instability of the bulk perovskite lattice. Therefore, reinforcing the perovskite lattice from within represents a pivotal yet underexplored route toward intrinsically robust devices.

Herein, inspired by the classical metallurgical concept of solid solution strengthening, we propose a solid-solution-induced lattice hardening (SSILH) strategy to directly reinforce the stiffness of perovskite films. We selected the lanthanum ion (La^3+^) as an optimal dopant due to its suitable ionic radius and strong halide bonding, therefore inducing lattice contraction and strengthening the chemical bonds throughout the entire framework. We demonstrate that the SSILH strategy effectively enhances perovskite stiffness, as quantified by the Young’s modulus. The more rigid lattice suppresses thermal expansion, increases defect formation activation energy, and raises barriers against ion migration and phase transitions. In addition, we employ a stable, hydrophobic p-dopant, 4-isopropyl-4′-methyldiphenyliodonium tetrakis(pentafluorophenyl) borate (TPFB), to construct a robust hole-transport layer (HTL). Finally, our n-i-p PSCs yield a champion efficiency of 26.54% and exceptional operational stability.

## RESULTS AND DISCUSSION

### Solid-solution-induced lattice hardening mechanism

The structural stability and mechanical robustness of perovskite (ABX_3_) are mainly governed by the strength of the pivotal B–X bonds that constitute the octahedral framework [30,31]. We therefore reason that alloying the B-site located at the octahedral center, which leads to solid solution formation, represents an effective strategy for tuning the intrinsic stiffness of perovskite. We illustrate the SSILH mechanism in Fig. 1a. Among potential lanthanide dopants for the B-site, we selected La^3+^ owing to its higher bond energy with iodine and its slightly smaller ionic radius compared with Pb^2+^ (Figs S1 and S2). The strengthening mechanism operates through two synergistic effects: (1) The intrinsically higher La–I bond energy locally reinforces the octahedral network. (2) The smaller ionic radius of La^3+^ introduces compressive strain (chemical pressure), leading to a uniform lattice contraction and shorter bond lengths. This contraction enhances atomic orbital overlap and increases the overall bond strength across the lattice (Fig. S3 and Table S1). The resulting denser and more rigid lattice directly enhances the intrinsic perovskite stiffness, which suppresses the B–X bond breaking, reduces point vacancies, and impedes ion migration.

**Figure 1.**
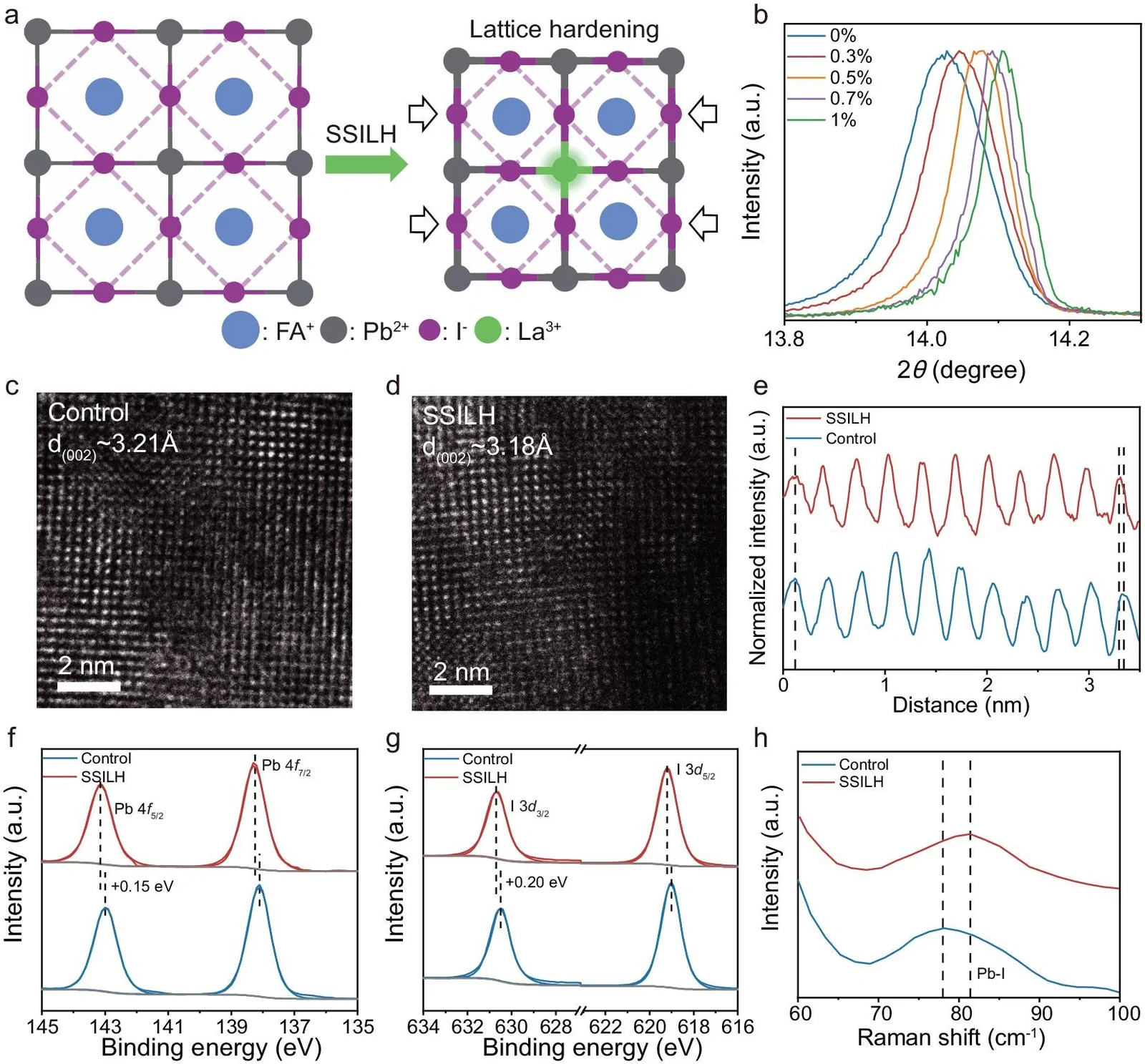
Characterization of the crystal structure and illustration of lattice hardening in perovskites. (a) Schematic illustration of the solid-solution-induced lattice hardening (SSILH) mechanism in FAPbI_3_ perovskite via La^3+^ doping (blue, organic cation; gray, lead ion; purple, iodine ion; green, lanthanum ion). (b) XRD of perovskite films doped with various amounts of LaI_3_. (c and d) High-resolution transmission electron microscopy (HRTEM) images of control (c) and SSILH (d) perovskite films. (e) The lattice plane spacing calculated along the intensity variation distribution within the range of 3.5 nm is recorded in the corresponding image. XPS spectra of (f) Pb 4*f*, (g) I 3*d* for control and SSILH perovskite films. (h) Raman spectra of the control and SSILH perovskite films. The characteristic peak observed at the dotted line corresponds to the vibration mode of the metal halide octahedron of the metal halide perovskite.

To confirm La^3+^ incorporation while excluding anion effects, X-ray diffraction (XRD) was performed on perovskite films doped with LaI_3_, as shown in Fig. 1b. With increasing La^3+^ concentration, the perovskite (001) diffraction peak gradually shifted to a larger diffraction angle, indicating lattice contraction due to the smaller radius of La^3+^ (106 pm) compared to the Pb^2+^ (119 pm). The optimum dopant concentration is around 0.5%, while further increasing the dopant concentration affects the crystallization and deteriorates the crystal quality of perovskite films (Figs S4 and S5). The crystal structures of control and SSILH perovskite films were further analyzed by the high-resolution transmission electron microscopy, which both display well-resolved lattice fringes (Fig. 1c–e). The average interplanar spacing of control perovskite corresponding to the (002) plane of an orthorhombic structure is 3.21 Å while that of SSILH sample is 3.18 Å. The energy dispersive spectrometer (EDS) mappings confirm the homogeneous distribution of La within the film (Fig. S6 and Table S2).

X-ray photoelectron spectroscopy (XPS) was performed on the control and SSILH perovskite films. It is found that the peaks of both Pb 4*f* and I 3*d* in the SSILH perovskite films shifted towards higher binding energies (Fig. 1f and g), indicative of a stronger Pb–I interaction due to the lattice contraction. The characteristic peaks of La 3*d* were detected, which further proved the presence of La ions in the SSILH film (Fig. S7). The Raman spectroscopy is extremely sensitive to lattice vibrations, and we tested the low-frequency vibrations that are predominantly associated with the octahedral skeleton to probe the change in bond strength [32]. The low-frequency modes around 80 cm^−1^ are assigned to Pb–I stretching vibrations [33]. As shown in Fig. 1h, the characteristic Pb–I stretching peak exhibits a distinct blue shift from 78.12 cm^−1^ in the control film to 81.39 cm^−1^ in the SSILH film. The shift reflects a higher vibrational frequency of the Pb–I bond, which is a direct spectroscopic signature of lattice contraction and enhanced bond strength.

### Stiffness and stability reinforcement of perovskite films

The lattice hardening directly improves the stiffness and stability of the perovskite films. We first tested the mechanical properties of perovskite through nanoindentation measurements (Fig. 2a and Table S3). The Young’s modulus, which reflects the intrinsic stiffness and bond strength of the material, increased from 27.96 GPa for the control film to 35.71 GPa for the SSILH film at a standard indentation depth of 100 nm. Correspondingly, the hardness of films increased from 0.70 GPa for the control to 0.91 GPa for the SSILH perovskite. We further probed mechanical properties of perovskite surface via quantitative nanomechanical mapping-atomic force microscope (QNM-AFM) measurements. The results showed that the average surface Young’s modulus increased from 5.24 GPa for the control film to 7.27 GPa for the SSILH film (Fig. 2b, c and Fig. S8). The consistent mechanical enhancement observed with nanoindentation and QNM-AFM measurements firmly verify that the SSILH strategy reinforces the lattice stiffness throughout the film. In addition, the increased grain size and intragrain Young’s modulus observed in the SSILH films rule out grain-boundary strengthening as the origin of the hardening (Fig. S3 and Fig. 2c).

**Figure 2.**
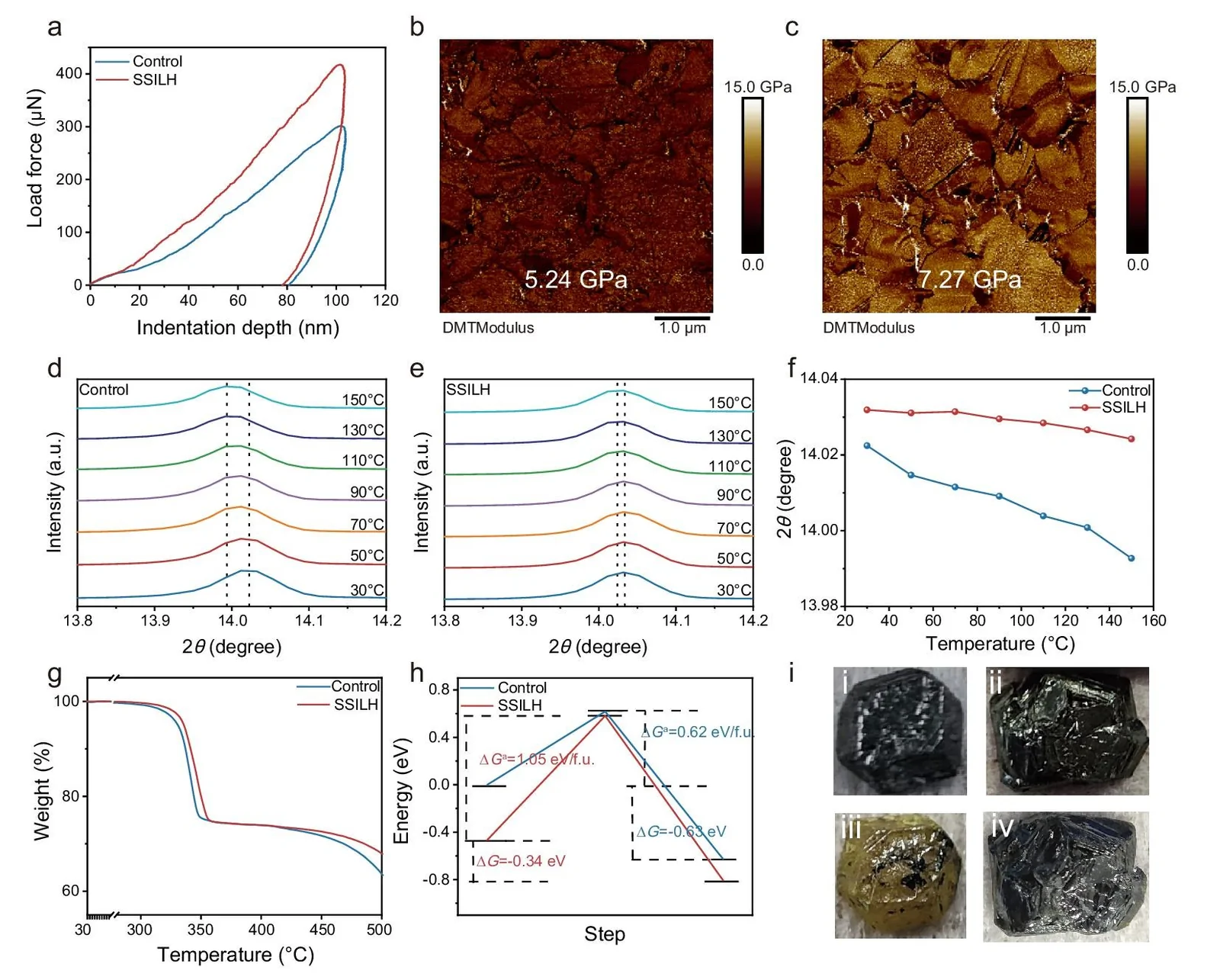
Enhanced mechanical robustness and stability of perovskite films. (a) Representative load force–dependent indentation depth curve from nanoindentation measurements on control and SSILH perovskite films at a maximum depth of 100 nm. (b, c) Quantitative nanomechanical atomic force microscopy (QNM-AFM) images of the control (b) and SSILH (c) perovskite films. (d, e) The *in-situ* X-ray diffraction (XRD) patterns of the control (d) and SSILH (e) perovskite films at different temperatures, highlighting the (001) peak shift. (f) Evolution of the (001) diffraction peak position (2*θ*) as a function of temperature for control and SSILH films, derived from (d) and (e). (g) Thermogravimetric analysis (TGA) curves for control and SSILH perovskite samples. (h) Calculated energy profiles (thermodynamic driving force and activation barrier) for the *α*-to-*δ* phase transition in control and SSILH FAPbI_3_. (i) The photographs of the control and SSILH perovskite single crystals after 100 days of aging (RH = 40%–50%, *T* = 25°C). i: the fresh control perovskite single crystals. ii: the fresh SSILH perovskite single crystals. iii: the control perovskite single crystals after aging. iv: the SSILH perovskite single crystals after aging.

We monitored the structural evolution of the perovskite thin films at different temperatures using *in-situ* XRD (Fig. S9). The samples were heated from room temperature to 150°C. As shown in Fig. 2d, the (001) diffraction peak of control perovskite film shifted progressively toward lower angles, corresponding to lattice expansion. Quantitative analysis reveals that the (001) interplanar spacing increases from 6.312  to 6.325 Å, representing a thermal expansion of 0.21%. In contrast, the SSILH film exhibits significantly suppressed thermal expansion, with the (001) interplanar spacing changing by only 0.08% under same conditions (Fig. 2e, f and Table S4). The calculated coefficient of thermal expansion along the (001) direction decreases from 17.5 × 10^−6^ K^−1^ for the control film to 6.67 × 10^−6^ K^−1^ for the SSILH film. The marked reduction in the thermal expansion coefficient can be attributed to the increased lattice stiffness, which effectively resists bond elongation and structural distortion during heating. The improved stability at the atomic scale suggests a higher energy barrier for thermally driven degradation, which can be reflected in the macroscopic thermal decomposition behavior. Indeed, the thermogravimetric analysis confirms that the SSILH perovskite sample undergoes FAI loss and structural decomposition at significantly higher temperatures compared to the control sample (Fig. 2g).

To study the effect of lattice hardening on perovskite phase stability, we calculated the *α*-to-*δ* phase transition pathway using an SSW pathway sampling method (Fig. S10). For control FAPbI_3_, the *α*-to-*δ* phase transition has a thermodynamic driving force (Δ*G*, energy difference between the *α* and *δ* phases) of −0.63 eV and an energy barrier (Δ*G*^a^) of 0.62 eV per formula unit (eV/f.u.). In contrast, SSILH perovskite exhibits a reduced thermodynamic driving force to −0.34 eV and a significantly increased activation barrier to 1.05 eV/f.u. (Fig. 2h). The phase transition pathway showed that the *α*-to-*δ* phase transition, which is governed by shear-driven rearrangement of the octahedra without long-range atomic diffusion, is critically hindered by the stronger B–X bond in the SSILH perovskite (Fig. S11). We grew the FAPbI_3_ perovskite single crystals and confirmed that the SSILH FAPbI_3_ single crystal has better α-phase stability in ambient air (RH = 40%–50%) compared to control crystal (Fig. 2i). Similarly, the SSILH perovskite films also show superior resistance to the *α*-to-*δ* transition under the same humid conditions, as evidenced by the XRD and ultraviolet-visible (UV-vis) absorption spectroscopy (Figs S12 and S13).

### Photovoltaic performance of PSCs based on stable HTLs

We conducted the density functional theory (DFT) calculations to study how lattice hardening affects the defect formation. By applying hydrostatic pressure to simulate the compressive strain induced by La^3+^ doping, we found that lattice contraction significantly increases the formation energy of various point defects, including V_I_, I_I_ and Pb_I_ (Fig. 3a). Concurrently, the activation energy for iodine ion migration, which proceeds via a vacancy-mediated pathway along the octahedral edge, is also improved with increasing pressure (Fig. S14). These calculations confirm that the hardened lattice not only thermodynamically suppresses the defect formation, but also kinetically hinders ion migration, providing a fundamental explanation for the enhanced photoelectric property and operational stability in the following. We performed the steady-state photoluminescence (PL) and PL mapping measurements for the control and SSILH films (Fig. S15 and Fig. 3b, c). The results showed that the SSILH films possess a much stronger PL intensity, indicating that the defect-induced non-radiative recombination was effectively inhibited. This is confirmed by time-resolved PL measurements, which show an increase in the average carrier lifetime from 2.3 μs for the control to 6.3 μs for the SSILH film (Fig. 3d). The UV-vis absorption measurements showed that SSILH has a negligible effect on the optical bandgap (Fig. S16). However, a notable decrease in the Urbach energy from 54.64 meV for control to 44.01 meV for SSILH film was observed (Fig. 3e). The reduction in band-tail states, indicating suppressed shallow traps and structural disorder [34], corresponds to the improved carrier lifetime and further validates the defect-inhibition effect of the SSILH strategy.

**Figure 3.**
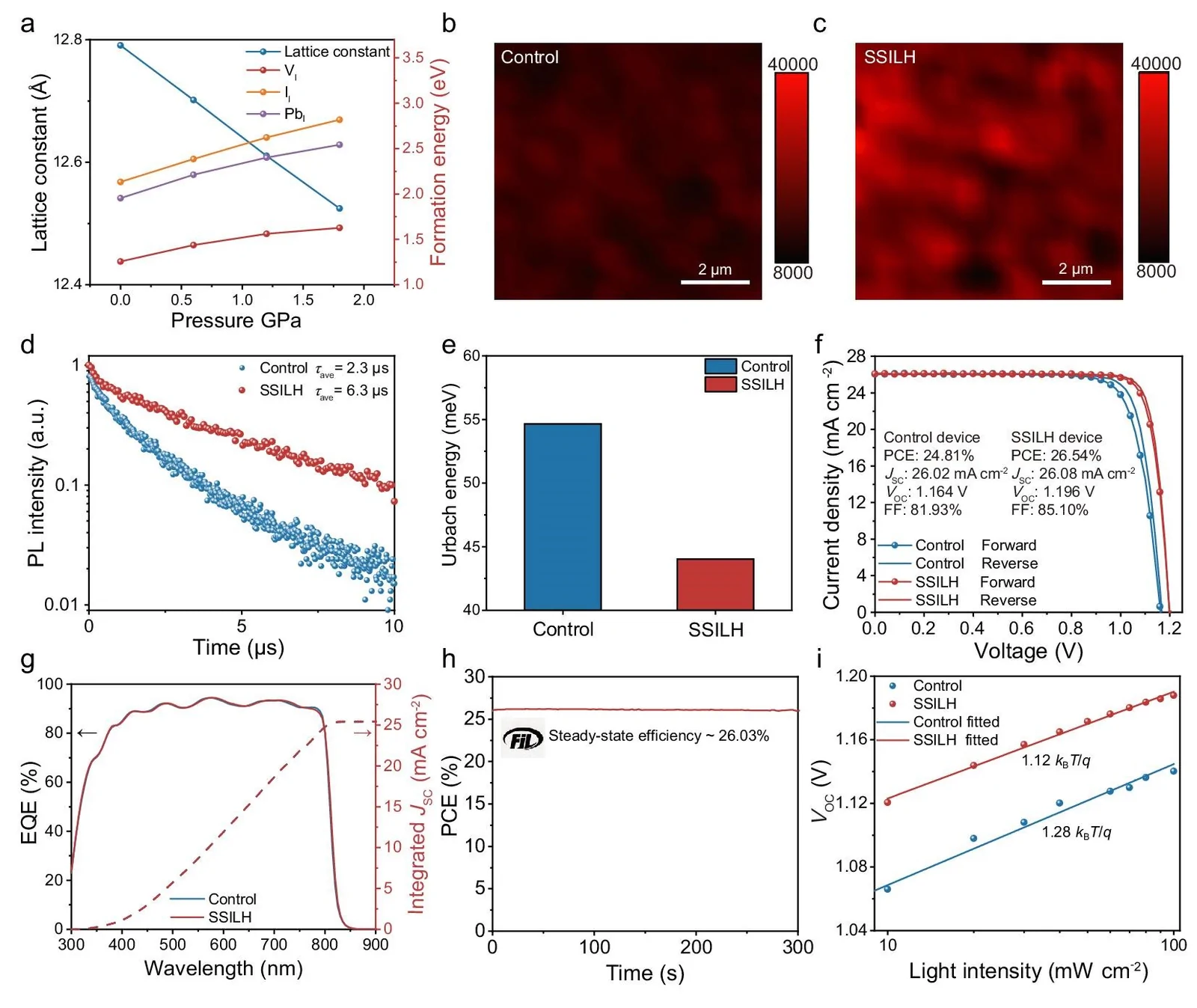
Optoelectronic properties and photovoltaic performance of PSCs. (a) Calculated evolution of the lattice constant and defect formation energies for key point defects as a function of hydrostatic pressure, simulating the effect of La^3+^-induced compressive strain. (b, c) Steady-state photoluminescence (PL) mappings of the control (b) and SSILH (c) perovskite films. (d) TRPL decay times of the control and SSILH perovskite films. (e) Urbach energy extracted from the sub-bandgap absorption tails of control and SSILH perovskite films. (f) *J–V* curves of the champion control and SSILH devices under forward and reverse scan. (g) EQE spectra and integrated *J*_SC_ of the control and SSILH devices. (h) Steady state efficiency power output of SSILH device for 300 s certified by NPVM. (i) Light intensity dependence of *V*_OC_ for the control and SSILH devices. The ideality factor was calculated from the slope of the linear fit of the semilogarithmic plot.

Recognizing that the stability of n-i-p PSCs is generally limited by the hole transport layer (HTL), we replaced the conventional and hygroscopic Li-TFSI/tBP with a non-volatile and hydrophobic salt, 4-isopropyl-4′-methyldiphenyliodonium TPFB as the p-type dopant for Spiro-OMeTAD (Figs S17 and S18). This p-dopant eliminates the need for oxygen-assisted oxidation, thereby simplifying the processing steps (Fig. S19). It also enables an HTL with a higher glass-transition temperature and superior thermal stability in comparison with Li-TFSI/tBP without compromising hole mobility, conductivity, and work function (Figs S20–S23).

With this stable HTL as a baseline, we fabricated the devices with an n-i-p structure of FTO/SnO_2_/perovskite/HTL/Au to study the effect of SSILH on the photovoltaic performance and subsequent stability. We separately used LaI_3_ and LaCl_3_ as the additives to dope the FA_0.94_Cs_0.06_PbI_3_ perovskite films. The statistical distribution of the measured efficiencies for the PSCs is shown in Fig. S24. The La dopant primarily improves the open-circuit voltage (*V*_OC_) and fill factor (FF), mainly due to the reduced defects as mentioned above. In addition, the LaCl_3_-based devices show better performance than those based on LaI_3_ dopant, which can be attributed to the Cl-assisted crystallization and defect passivation [35]. Thus, the LaCl_3_ dopant was used to achieve high performance of devices and the doping concentrations of TPFB were carefully optimized (Fig. S25). The typical current density-voltage (*J–V*) curves of the champion control and SSILH devices and corresponding parameters are shown in Fig. 3f and Table S5. The maximum power conversion efficiency (PCE) of the control device is 24.81% with a short-circuit current density (*J*_SC_) of 26.02 mA cm^−2^, an *V*_OC_ of 1.164 V and a FF of 81.93% under reverse scanning. In comparison, the SSILH device yielded a PCE of 26.54% with a *J*_SC_ of 26.08 mA cm^−2^, an *V*_OC_ of 1.196 V and a FF of 85.10% under reverse scanning. The SSILH device exhibits a reduced hysteresis index (Table S5). The integrated *J*_SC_ values for the external quantum efficiency (EQE) of the control and SSILH devices are 25.39 and 25.45 mA cm^−2^, respectively (Fig. 3g). One SSILH device was measured by the independently accredited testing center (National PV Industry Measurement and Testing Center, NPVM), achieving a certified efficiency of 26.35% with a steady-state efficiency of 26.03% (Fig. 3h and Fig. S26), which ranks among the highest reported efficiencies for n-i-p PSCs generally using tBP/Li-TFSI doped Spiro-OMeTAD (Table S6). The same SSILH strategy was extended to inverted devices, confirming the generality of the approach (Fig. S27).

We calculated the relationship between *V*_OC_ and light intensity (Fig. 3i and Fig. S28), where the value of ideality factor (*n*) can reflect the trap density of solar cells. The slope of the fitting curve of the SSILH device is 1.12*k*_B_*T*/*q*, which is much lower than that of the control devices (1.28*k*_B_*T*/*q*). This reduction indicates a lower contribution of trap-assisted recombination in the SSILH solar cell. We used the space-charge-limited current (SCLC) measurement of hole-only device to analyze the defect density of perovskite films. The calculated trap density (*n*_trap_) of the SSILH sample is 3.53 × 10^15^ cm^−3^, which is lower than that of the control sample (5.18 × 10^15^ cm^−3^, Fig. S29).

### Device stability

We systematically evaluated the operational stability of the devices under continuous illumination and damp-heat stress. For stability testing, the thin PCBM and MoOx films were introduced as the buffer layers at the SnO_2_/perovskite and HTL/Au interfaces, respectively, to improve the interfacial stability. Under maximum power point (MPP) tracking at 40 ± 5°C and 1-sun illumination, the SSILH device showed negligible efficiency decay over 1800 h (Fig. 4a). In comparison, the control device retained >85% of its initial efficiency under the same conditions. When the operating temperature was increased to 65°C, the stabilizing effect of the hardened perovskite lattice became even more pronounced. The SSILH device maintained >95% of its initial PCE after 1000 h of continuous operation, while the control device lost >30% within 600 h (Fig. 4b). Damp-heat testing (85°C/85% relative humidity, following IEC 61215:2016) further confirmed the superior stability of the SSILH device. After 1000 h, the encapsulated SSILH devices retained 90% of their initial efficiency, in contrast to ∼70% for the control devices (Fig. 4c).

**Figure 4.**
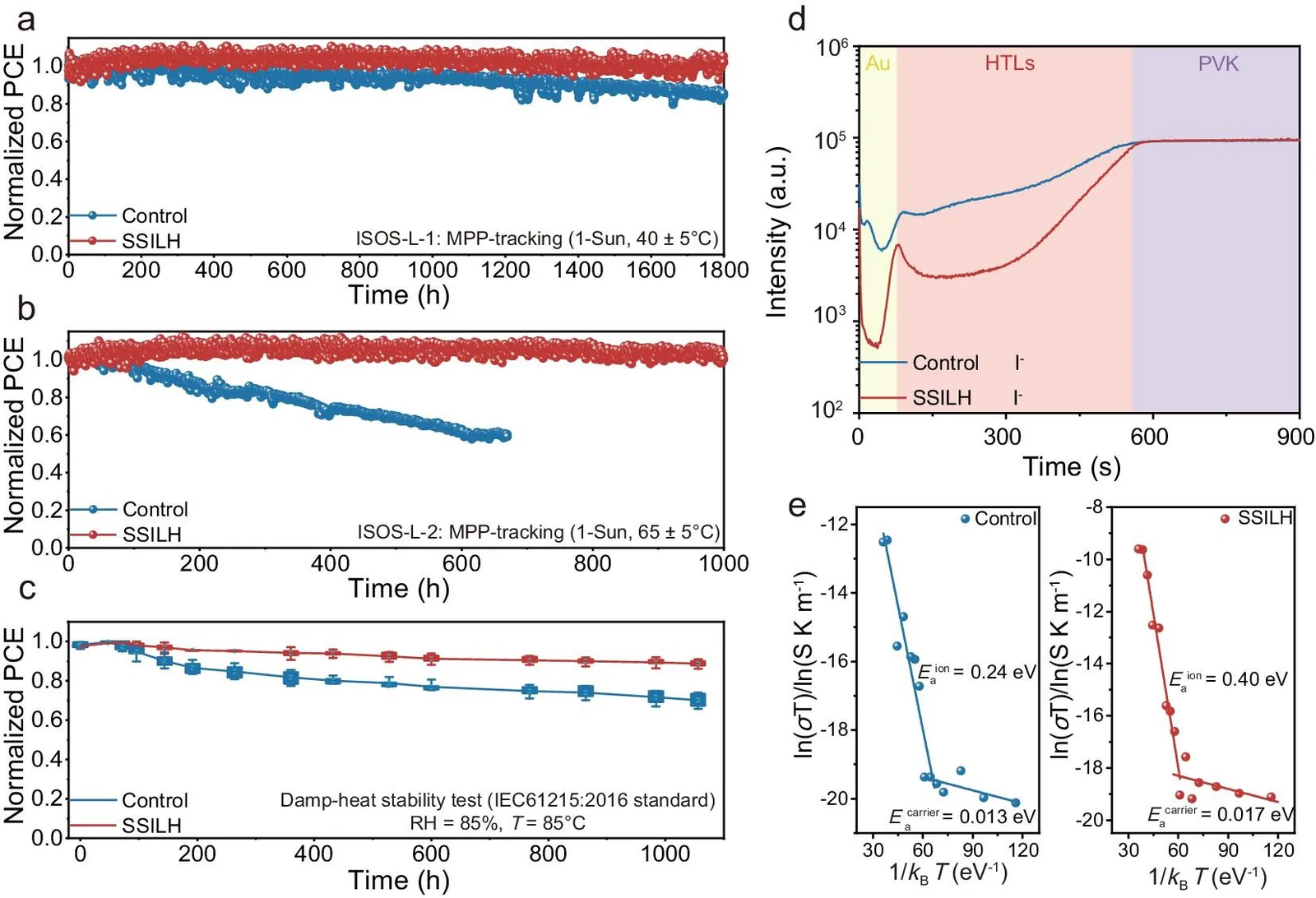
Long-term operational stability of devices based on TPFB-doped HTLs. (a) The MPP tracking of encapsulated control and SSILH devices under continuous 1-sun illumination in N_2_ at 40 ± 5°C. The initial PCEs of the control and SSILH devices used for the stability tests were 23.07% and 24.14%, respectively. (b) The MPP tracking of encapsulated control and SSILH devices in N_2_ at 65 ± 5°C. The initial PCEs of the control and SSILH devices used for the stability tests were 23.03% and 24.06%, respectively. (c) The evolution of the PCEs of encapsulated devices tracked under a relative humidity of 85% at 85°C. (d) The TOF-SIMS of the iodine element distributions in the control and SSILH devices after long-term operational stability test. (e) Temperature-dependent conductivity measurements of the control and SSILH perovskite films.

To understand the origin of this enhanced stability, we analyzed the aged devices using time-of-flight secondary ion mass spectrometry. Depth profiling revealed severe accumulation of iodine in the HTL and Au layers of the control device, indicative of pronounced I^−^ migration under operational stress (Fig. 4d). In contrast, the I^−^ signal in these layers was approximately an order of magnitude lower for the SSILH device, demonstrating effective suppression of halide migration. This finding was corroborated by XPS analysis of aged electrode surfaces, which showed a lower iodine concentration for the SSILH devices (Fig. S30).

The activation energy (*E*_a_) for ion migration was quantified via temperature-dependent conductivity measurements according to the Nernst-Einstein relation [36]: *σT* = *σ*_0_ × exp(−*E*_a_/*k_B_T*); where *σ* is ionic conductivity, *σ*_0_ is a constant, *E*_a_ is the activation energy for ion migration, *k*_B_ is Boltzmann’s constant and *T* is the temperature. The *E*_a_ values of the control and SSILH films derived from the slope of ln(*σT*) to 1/*k*_B_*T* were 0.24 and 0.40 eV. This substantial increase in activation energy confirms that the SSILH strategy creates a higher energy barrier for ion migration (Fig. 4e), in agreement with our previous simulation result. Furthermore, EDS of films aged at 85°C for 21 days showed that the I:Pb stoichiometric ratio remained close to 3 for the SSILH film but decreased significantly for the control film (Fig. S31). This indicates that the strengthened B–X bonding in the hardened lattice effectively inhibits the volatilization of iodine species, thereby preserving the compositional and structural integrity of the perovskite under thermal stress.

## CONCLUSION

In summary, we have developed a solid-solution-induced lattice hardening strategy to fundamentally ease the intrinsic soft-lattice instability of perovskite materials. This work establishes lattice stiffness as a quantifiable and targetable material property that determines optoelectronic performance and device longevity. By employing La^3+^ to form a solid solution, we achieved a denser and mechanically reinforced perovskite framework due to the compressive strain and enhancement of chemical bonds. It obviously increases the perovskite stiffness, yielding numerous benefits, including reduced harmful thermal expansion, higher activation energy for ion migration, and increased energy barrier for phase transitions. We also engineered the device stability from the charge-transport side by employing a thermally stable, hydrophobic p-dopant for the HTL, relieving the instability associated with conventional dopants. The synergy between intrinsically stiffer perovskite film and stable charge extraction yields n-i-p PSCs with a certified steady-state efficiency of 26.03% and exceptional operational stability. Our study provides a material-strengthening strategy and pioneers a mechanical-property-oriented design principle for developing efficient and inherently stable perovskite optoelectronic devices.

## Supplementary Material

nwag484_Supplemental_File

## Data Availability

All data are available in the main text or the supplementary materials. Other data that support the findings of this study are available from the corresponding author upon reasonable request.
