# Supplementary material for: Solid-solution-induced lattice hardening reinforces perovskite stiffness for stable solar cells
Source: Natl Sci Rev. 2026 Aug 25;13(19):nwag484. doi: 10.1093/nsr/nwag484 (PMC13725829; doi:10.1093/nsr/nwag484)
Supplement: Supplementary file 1 [file nwag484_supplemental_file.pdf]

Supplementary Information

Solid-solution-induced lattice hardening reinforces perovskite stiffness for stable solar cell

Jiahao Liang[1,2,3,†], Hailin Li[1,2,†], Tian Chen[1,2,†], Shengcai Zhu[1], Hepeng Wang[1,2], Zhouti Wang[1,2], Yuecheng Hu[1,2], Huahui Chen[1,2], Wenchang Huo[1,2], Ming Fang[3,*], Wenjun Liu[3], Hao Lin[1,2], Jiancan Yu[1,2], Jiangsheng Xie[1,2,*] and Pingqi Gao[1,2,4,*]

[1]School of Materials, Shenzhen Campus of Sun Yat-sen University, Shenzhen 518107, China;

[2]Institute for Solar Energy Systems, Guangdong Engineering Technology Research Center for Sustainable Photovoltaic Technology and Equipment, State Key Laboratory of Optoelectronic Materials and Technologies, Sun Yat-sen University, Guangzhou 510275, China;

[3]College of Materials Science and Engineering, Shenzhen University, Shenzhen 518060, China and [4]Sichuan Yat-sen Innovation Center of Photovoltaic Industry, Yibin 644609, China

***Corresponding authors.** E-mails: m.fang@szu.edu.cn; xiejsh8@mail.sysu.edu.cn; gaopq3@mail.sysu.edu.cn

[†]Equally contributed to this work.

The PDF file includes:

Supplementary Text

Figure S1 to S30

Table S1 to S5

References

Methods

Materials

Lead iodide (PbI_2, 99.99%, trace mentals basis), 4-Isopropyl-4'-methyldiphenyliodonium Tetrakis(pentafluorophenyl)borate (TPFB, >98%), (4(3,6-Dimethyl-9H-carbazole-9-yl) butyl) phosphonic acid (Me-4PACz, 99.0%) and bathocuproine (BCP >99%) were purchased from Tokyu Chemical Industry. $SnCl_2 \cdot H_2O$ (99.999%), urea (99.5%), thioglycolic acid (98%) and all solvents involved in the experiment (including DMF, NMP, IPA, CB, ACN) were purchased from Sigma-Aldrich. Bis(trifluoromethane) sulfonimide lithium salt (Li-TFSI, 99.95%), 4-tert-butylpyridine (*t*BP), Cesium iodide (CsI, 99.99%), methylamine hydrochloride (MACl, 99.9%), 2-(3-Fluorophenyl)ethanamine hydroiodide (m-F-PEAI, 99%), formamidinium iodide (FAI, 99.9%), 2,2',7,7'-Tetrakis [N, N-di(4-methoxyphenyl) amino]-9,9′-spirobifluorene (Spiro-OMeTAD, 99.9%), C60 (99%) were purchased from Xi'an Yuri Solar Co. 3,3'-(2,7-Dibromo-9H-fluorene-9,9-diyl)bis(N,N-dimethylpropan-1-amine) (FNBr, >98%) was purchased from Lumtec. Lanthanum iodide (LaI_3, 99.9%), Lanthanum chloride ($LaCl_3$, 99.9%) were purchased from Beijing Vokai Biotechnology Co. The above reagents were used without additional purification.

Device fabrication

FTO glass was successively cleaned by detergent, deionized water, acetone and ethanol. Before use, the FTO was treated under ultraviolet ozone for 20 min. The SnO_2 layer was deposited on the FTO substrate using chemical bath deposition technique. The solution was prepared by mixing urea (1250 mg), HCl (1250 μL), thioglycolic acid (25 μL) and $SnCl_2 \cdot 2H_2O$ (275 mg) into DI water (100 mL). The as-cleaned FTO glass was soaked into the solution for 4 h at 90°C. Then it was washed by deionized water and ethanol, dried by gas gun blowing, and followed by 170°C annealing for 1 h. After cooling to room temperature, the substrates were treated by UV-ozone for

15 min, followed by spin-coating 10 mM KCl in deionized water at 4000 rpm for 30 s and annealing at 150°C for 25 min. Before spin coating perovskite, the substrate was treated by an ultraviolet ozone cleaner for 15 min. After that, 40 μL perovskite precursor solution (1.58 M $FA_{0.94}Cs_{0.06}PbI_3$ with 30 mol% MACl, 0.5 mol% LaI_3 or $LaCl_3$ and excess 7.5 mol% PbI_2 in a mixture of DMF: NMP (5:1), the precursor solutions were stirred at 55˚C for 2 h and filtered using a 0.22 μm polytetrafluoroethylene membrane before use) was spin coated on the SnO_2 substrate at 4000 rpm for 70 s. The film was annealed at 100°C for 60 min in the air with ~30% RH. Then, 3 mg mL^{-1} m-F-PEAI solution was spin-coated on the perovskite films at 5000 rpm for 30 s. After that, the Spiro-OMeTAD solution (72.3 mg mL^{-1} in CB) with 2 mg TPFB and 30 μL FNBr (15 mg mL^{-1} in CB) was spin-coated on the perovskite film at 3000 rpm in N_2 glovebox. The devices were stored and aged in N_2 without needing any oxygen-assisted oxidation processes. Finally, 100 nm Au electrode was deposited by a thermal evaporation at a rate of 0.1-1.0 Å s^{-1} under the vacuum (<2.5 × 10^{-4} Pa) to complete the device fabrication process. For the operational stability test, the PCBM solution (3 mg mL^{-1}) was spin coated on the SnO_2 films at 3000 rpm for 30 s and 8 nm MoOx layer was deposited by thermal evaporation on the Spiro-OMeTAD.

For the p-i-n deviced, the Me-4PACz (0.5 mg ml^{-1} dissolved in ethyl alcohol) was spin-coated onto the precleaned FTO substrate at 3000 rpm for 30 s, then annealed at 100℃ for 10 min. The PDAI2 (1 mg mL^{-1} in IPA) was then spin-coated onto the perovskite films and annealed at 100℃ for 5 min. After cooling to ambient temperature, 25 nm C60 and 7 nm BCP were subsequently deposited by thermal evaporation, respectively. Finally,100 nm Ag electrode was evaporated.

Long-term stability test

The perovskite devices for stability test were encapsulated with polyisobutylene (HelioSeal PVS101, KÖMMERLIN) as a barrier layer and a small glass coverslip by a vacuum laminator.

The stability test at the continuous maximum power point tracking under 1 sun illumination (LED lamp) for the encapsulated PSCs was performed under N_2 atmosphere, without the use of any cooling mechanisms. The high-temperature operational stability tests were performed by tracking MPP of encapsulated PSCs under continuous 1 sun illumination at 65°C in N_2. For the damp-heat tests, encapsulated devices were aged in a damp-heat chamber at constant temperature and relative humidity levels (85°C and 85%), and measured periodically by *J-V* scans after cooling of devices.

Device characterization

For all devices, photovoltaic performance was measured by masking on the active area with a metal mask. *J-V* curves were extracted with a source meter (Keithley 2400) and a solar simulator (Enlitech SS-X) with 100 mW cm^{-2} illumination (AM 1.5G) and a calibrated silicon reference cell (Enlitech, KG2). The *J-V* measurements were carried out in N_2 atmosphere with forward (0-1.3 V) and reverse (1.3-0 V) scan modes (voltage steps of 20 mV and a delay time of 10 ms) at room temperature (~25°C). The active area was defined by a metal mask (0.0683 cm^2). The EQE measurements were performed in ambient air at ~25°C and ~35% relative humidity in the dark chamber using a PVE300-IVT210 system (Industrial Vision Technology (s) Pte Ltd) with monochromatic light focused on a device. Pulsed-voltage space-charge-limited current (SCLC) was recorded on Keithley 2400 meter. For the hole-only device, the bias voltage was increased from 0 to 4 V, with the voltage pulse width set to 20 ms and the time between voltage pulses set to 10 ms.

Film characterization

The XRD patterns of perovskite film were obtained by Bruker D8 ADVANCE. In-situ X-ray diffraction spectra were obtained by Bruker D8 ADVANCE with medium and low-temperature in-situ accessories. The nanoindentation measurements on perovskite films at a maximum depth

of 100 nm were performed using a Bruker TI 950 nanoindenter. UV-vis absorption spectrum was measured by UV-3600Plus (Shimadzu). XPS measurements were performed by Thermo Fisher Scientific. Scanning electron microscopy (SEM) was performed on a JEOL-JSM-6330F system. High-resolution transmission electron microscopy (HRTEM) was performed on a JEOL-F200 system with an acceleration voltage of 200 kV. The energy spectrum model is the JED-2300 series. Raman spectra were collected by a confocal micro-Raman spectrometer (inVin Renishaw), which is equipped with a 50x objective lens and a 532 nm laser. Steady-state PL and time-resolved PL spectra were measured by FLS1000 Series of Fluorescence Spectrometers with 450 nm pulse laser (VPL450 nm 85 mW). All the films were deposited on glass. For PL mapping images, galvo-based scanner was extra equipment. QNM-AFM and KPFM measurements were performed on an atomic force microscope (Dimension XR). A gold sample was used to calibrate the work function of Pt/Ir coated silicon tip before measurement.

Ion conductivity measurement

Variable temperature IV measurement was carried out by Lake Shore TTPX from 100 K-320 K. The ionic conductivity (σ) was extracted from the current response of a lateral perovskite device, with a configuration of Au/perovskite (100 μm)/Au, under an applied electrical field. The variable temperature IV measurement was carried out by Lake Shore TTPX in N_2 atmosphere with the working temperature controlled by a probe station (Lakeshore PS-100) from 100 K to 320 K.

Time-of-flight secondary ion mass spectrometry (TOF-SIMS) measurement

The depth distribution of ions in perovskite devices after MPP aging was measured by TOF-SIMS spectroscopy. The samples were analyzed using a TOF-SIMS V instrument (IONTOF) with a Bi^+ primary beam (30 keV and 1 pA) and Cs^+ sputter beam (2 keV and 1 nA). The sputter size was 350 μm × 350 μm and the analysis area was100 μm × 100 μm (for depth profiling).

Density functional theory (DFT) calculations

First-principles calculations based on density functional theory (DFT) were carried out using the Vienna Ab initio Simulation Package (VASP) with the version 6.4.2. The generalized gradient approximation (GGA) of Perdew-Burke-Ernzerhof (PBE) functional was employed as the exchange-correlation functional. The plane-wave cutoff energy of 400 eV was used. The ground-state atomic geometries were optimized by relaxing the force below 0.05 eV/Å and the convergence criteria for energy was set with the value of 1.0×10^{-5} eV/ell. We constructed a 2×2×2 $FAPbI_3$ supercell. The Brillouin zone sampling was performed with 2×2×2 k-points meshes. For the simulation of defects formation energy (E_f), the E_f were calculated using the following formula:

$$E_f = E_d^q - E_0 + \sum_i n_i \mu_i + q(E_F + E_{VBM}) + E_{corr}$$

Here, E_d^q and E_0 represent the energy of defected lattice with q charge state and energy of pristine lattice respectively. n_i and μ_i are the number and chemical potential of the varied ions during the defect formation. E_F , E_{VBM}, E_{corr} is the Fermi energy, energy of the valance-band maximum, and the energy correction due to finite supercell sizes. In order to reduce the amount of calculation, the calculation of defects in this paper is based on neutral defects.

Pathway sampling

We utilized the SSW pathway sampling method to explore the phase transition pathway of α-phase to δ-phase. In the SSW method, the movement on the potential surface was guided by the random soft mode (second derivative) direction, which is capable to explore potential energy surface exhaustively and unbiasedly. The pathway sampling was carried out in a 72-atom supercell, and more than 100 pairs of initial state /final state (IS/FS) were collected in DFT level. Based on the IS/FS pairs datasets, the pathways connecting the initial structure and the final structure can be determined in an atom-to-atom correspondence, then the transition state can be seamlessly located

by using the variable-cell double-ended surface walking approach (VC-DESW). After transition state searching, the lowest energy barrier pathways can be determined by sorting the energy barriers.

Synthesis of La-Doped α-$FAPbI_3$ single crystals

The single crystals were synthesized by the inverse temperature crystallization (ITC) method [1]. A 1.0 M $FAPbI_3$ perovskite precursor solution was prepared by dissolving equimolar formamidinium iodide and PbI_2 in γ-butyrolactone. The amounts of LaI_3 (0.5%, 1.0%) in the perovskite solution were calculated according to the molar ratio with Pb. After stirring at 60°C overnight, the solution was filtered through a 0.22 μm polytetrafluoroethylene (PTFE) filter and transferred into glass vials. The filtered solution was heated at 120°C for 2 h to obtain seed crystals. Large single crystals were obtained by placing the seed crystals in fresh precursor solution and allowing them to grow further at 100°C. After being taken out of the solution, the resulting large crystals were washed with cyclohexane to remove unreacted chemicals and subsequently dried at 150°C for 1 h in a vacuum chamber. All crystals were stored in an N_2 glove box before use.

Analysis of Fig. S27

Light-intensity-dependent V_{OC} analysis is essential to decouple trap-assisted recombination from radiative losses. For perovskite solar cells, V_{OC} is a function of short-circuit current (J_{SC}), and J_{SC} is in direct proportion to light intensity ($J_{SC} \propto I_{\alpha}$) [2].

$$V_{OC} = \frac{nk_B T q^{-1}}{\ln(\frac{J_{SC}}{J_0} + 1)}$$

Where n, k_B, T, q, J_0 are the ideality factor, Boltzmann's constant, working temperature, basic charge, and saturation current density (in the dark). For devices with no trap-assisted single-molecule recombination, the value of n is close to 1. With comparison of a slope of $1.28k_BT/q$ in

the control device, a reduced slope of 1.12k_BT/q in the SSILH device was observed, indicating suppressed trap-mediated recombination.

Analysis of Fig. S28

The *J-V* curves of the hole-only devices were obtained using a Keithley 2400 Source Meter under dark conditions. The defect density of the control and SSILH films was measured by the SCLC model, using a hole-only structure of FTO/PEDOT:PSS/Perovskite/Spiro-OMeTAD/Au and taking current-voltage to a space-charge-limited form [3].

$$n_{trap} = \frac{2\varepsilon_0 \varepsilon V_{TFL}}{\mathrm{e}L^2}$$

Where ε_0 is the vacuum permittivity, ε is the relative dielectric constant of perovskite, and L is the perovskite thickness. V_{TFL} is the trap-filled limit voltage. n_{trap} is the trap density.

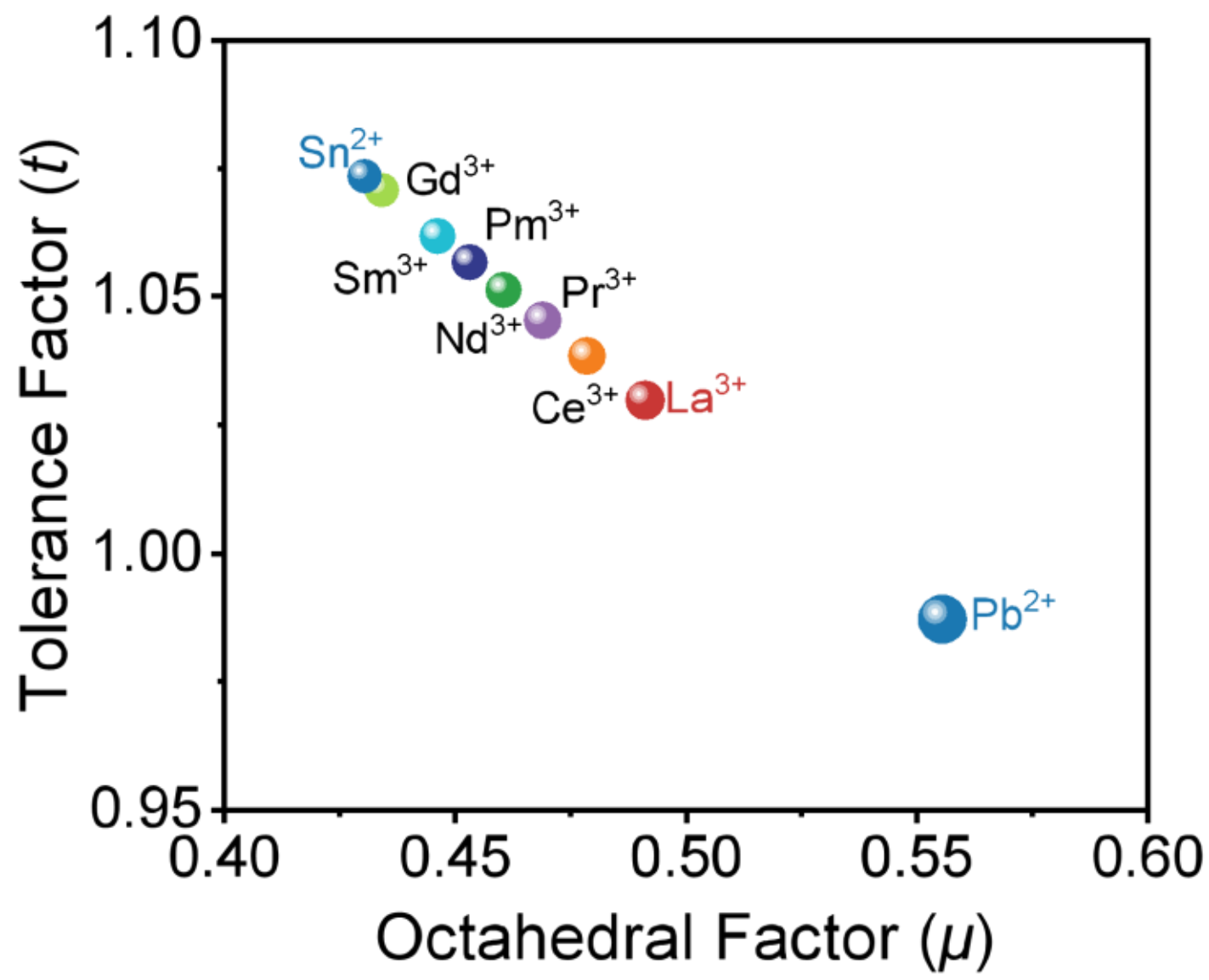

Figure S1 | Calculation of Tolerance factors (t) and Octahedral factor (μ) for lanthanide ions, Sn^{2+} and Pb^{2+}.

The stability of perovskite structure (ABX_3) generally requires an appropriate Goldschmidt tolerance factor $t = \frac{r_A + r_B}{\sqrt{2}(r_B + r_X)}$ and octahedral factor $\mu = \frac{r_B}{r_X}$, where r_A, r_B, and r_X are the ion radii of the A, B, and X positions, respectively [4]. Based on the typical volcano-type relationship, the optimal value μ for the stable perovskite phase would be around 0.5 [5], suggesting that La (μ = 0.48) should be the most suitable ion for B-site substitution.

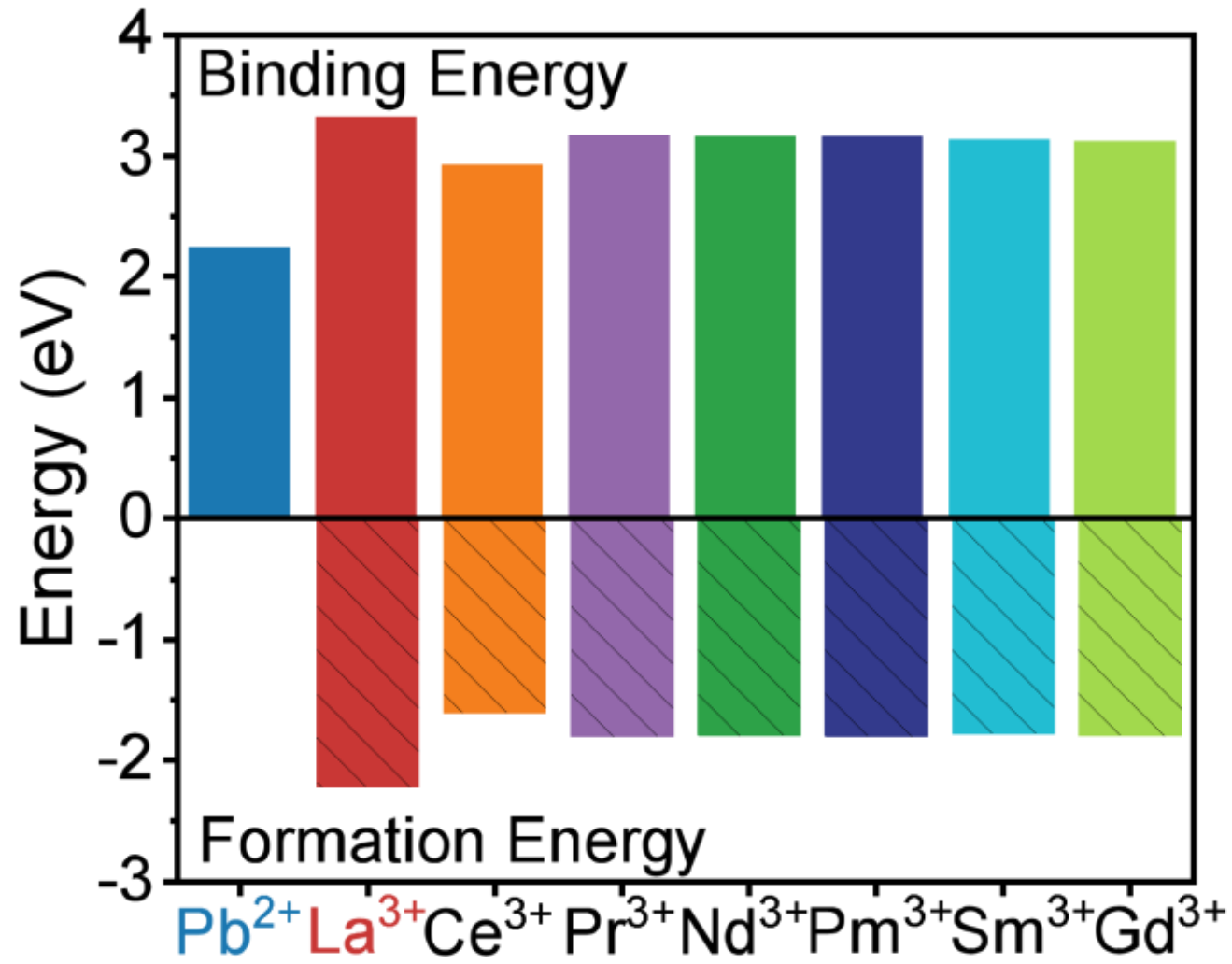

Figure S2 | Calculation of formation energies (shadow area) and B-X binding energies (solid color area) of different lanthanide ions.

The La ion possesses the lowest formation energy among all lanthanide ions, and leads to the strongest bond energy of La-I, which is much larger than that of Pb-I.

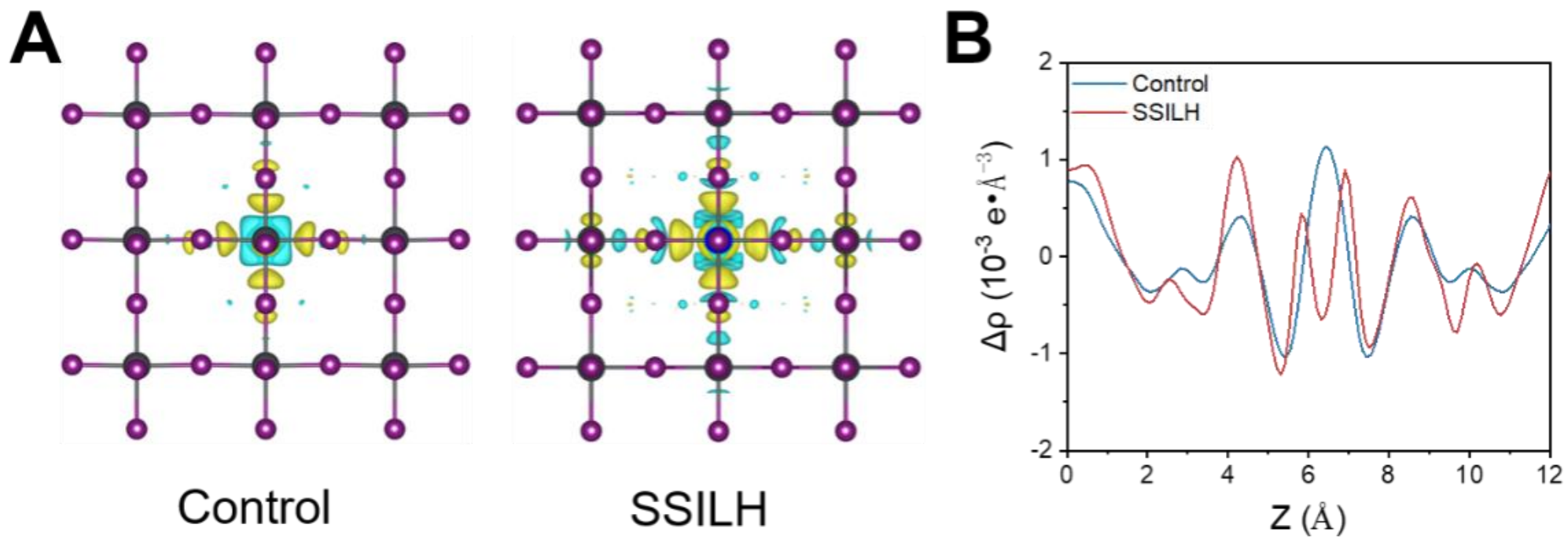

Figure S3 |

(A) The differential charge density diagram of $FAPbI_3$ (control) and La-doped $FAPbI_3$ (SSILH).

(B) The plane-averaged charge density difference of $FAPbI_3$ (control) and La-doped $FAPbI_3$ (SSILH). The reference coordinate axis is the Z-axis.

The simulation results show that SSILH not only enhances the distribution of the electron cloud in the central BX_6 octahedron, but also enhances the distribution of the electron cloud in the surrounding PBI_6. Compared with the control, the SSILH introduces isotropic compressive strain, resulting in lattice compression. This contraction enhances the orbital overlap of the space electron cloud and leads to an increase in bond energy. This also explains the solid solution-induced lattice hardening.

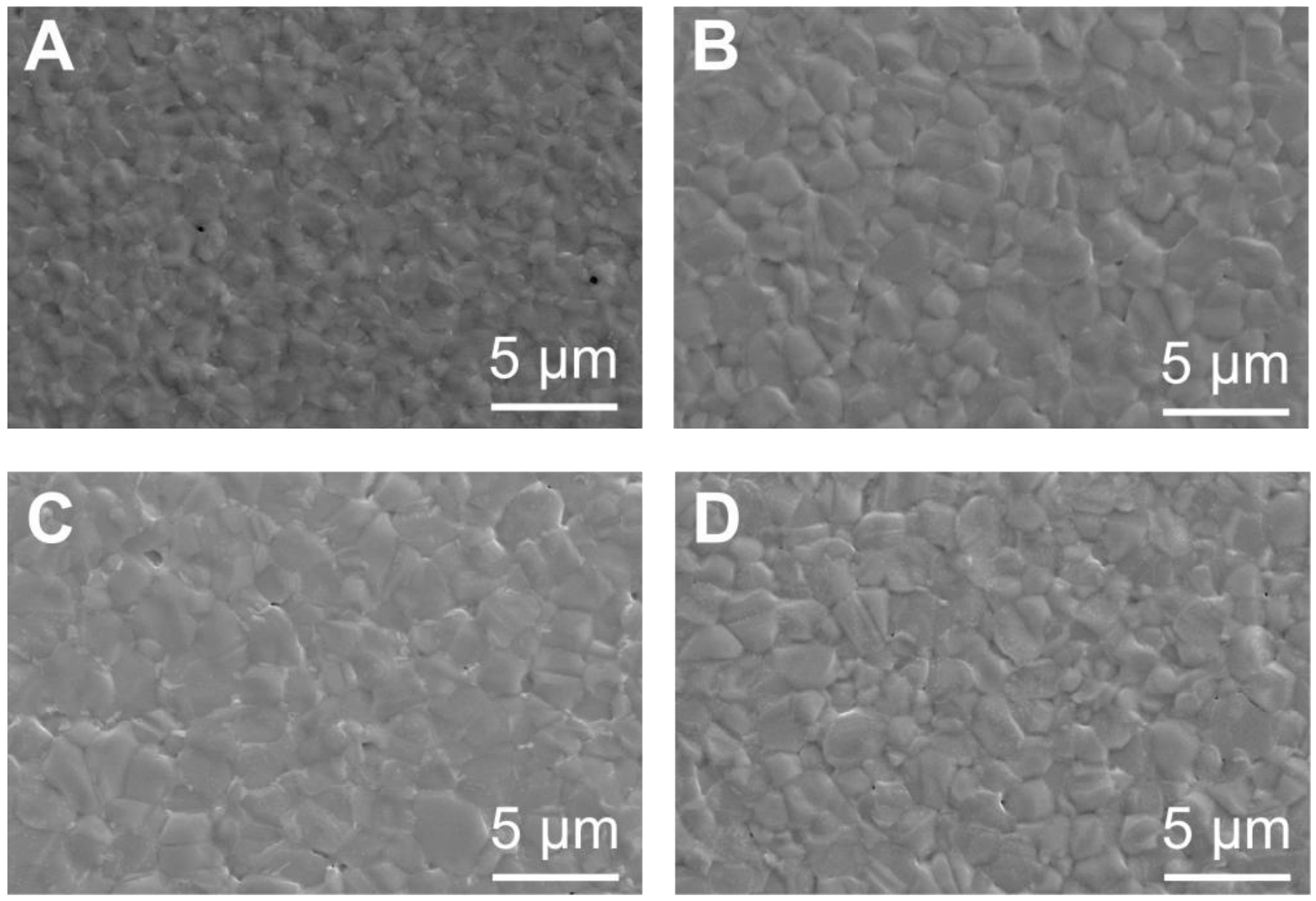

Figure S4 | Morphological analysis of perovskite.

Top-view SEM images of the perovskite films with different doping concentrations of La^{3+}. (**A**) 0%, (**B**) 0.3%, (**C**) 0.5%, and (**D**) 0.7%.

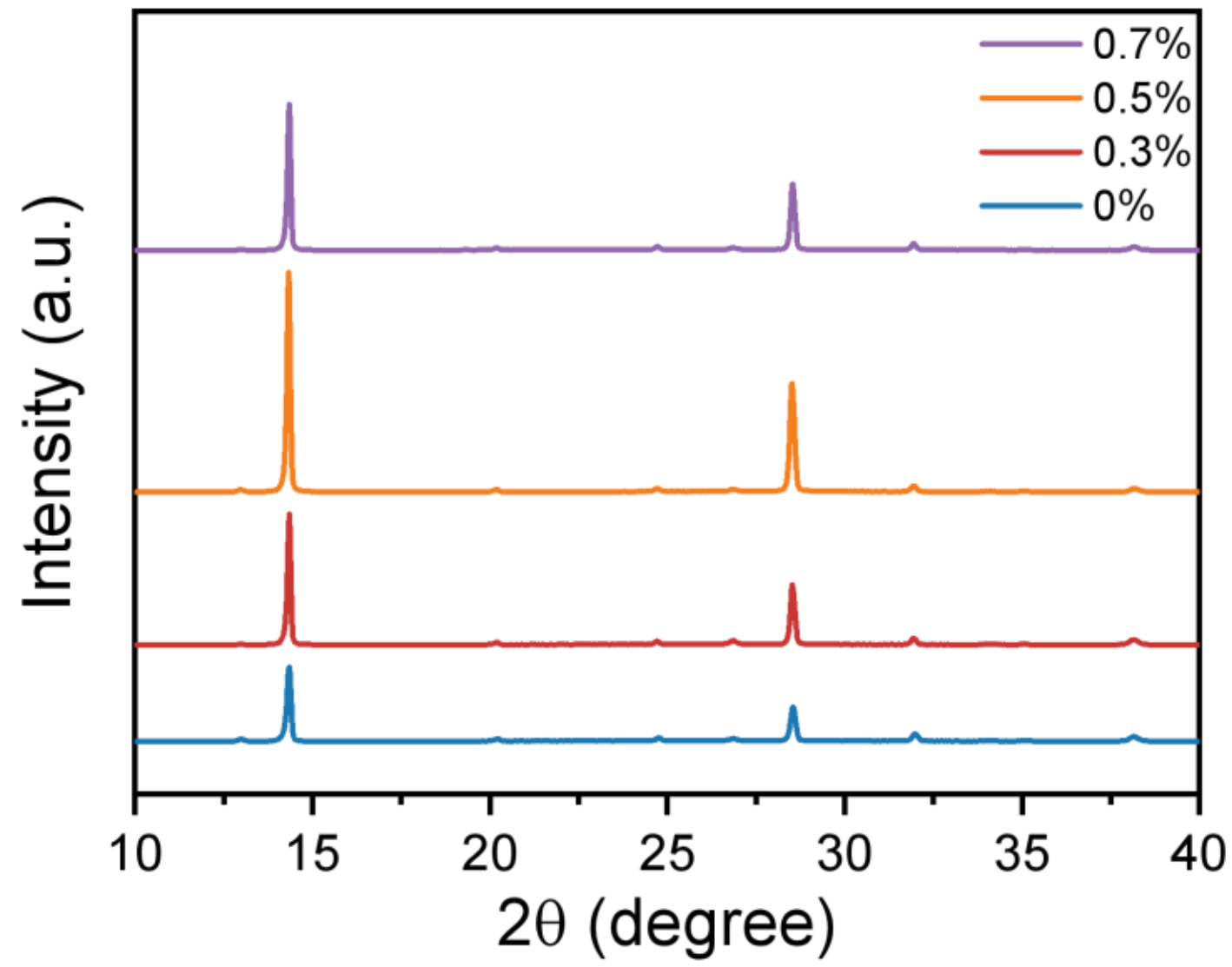

Figure S5 | Crystal structures analysis of perovskite.

XRD patterns of the perovskite films with different doping concentrations of La^{3+}.

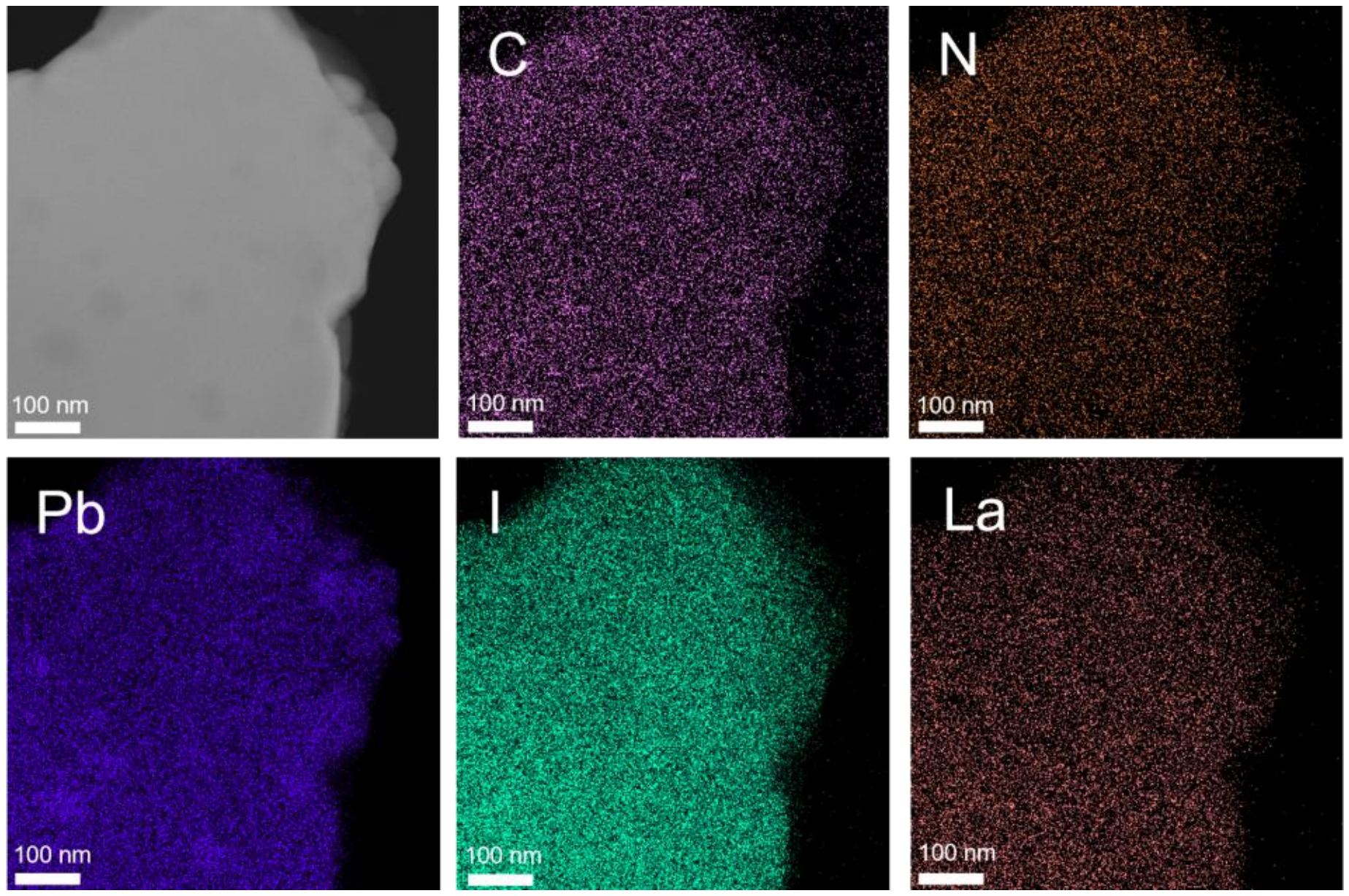

Figure S6 | Element distribution characterization.

EDS mapping of the SSILH perovskite films.

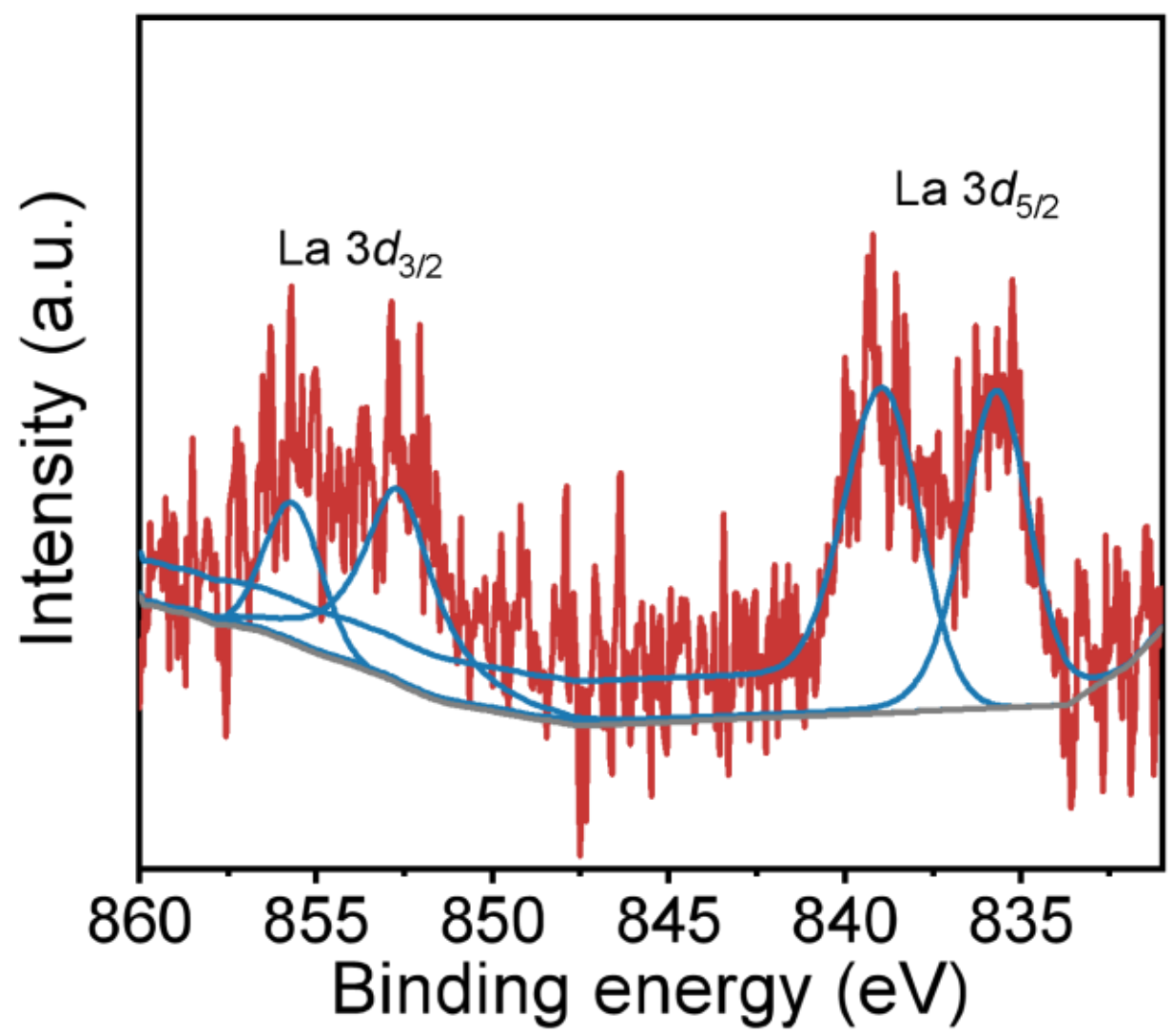

Figure S7 |

XPS spectra of La 3*d* for the SSILH perovskite films.

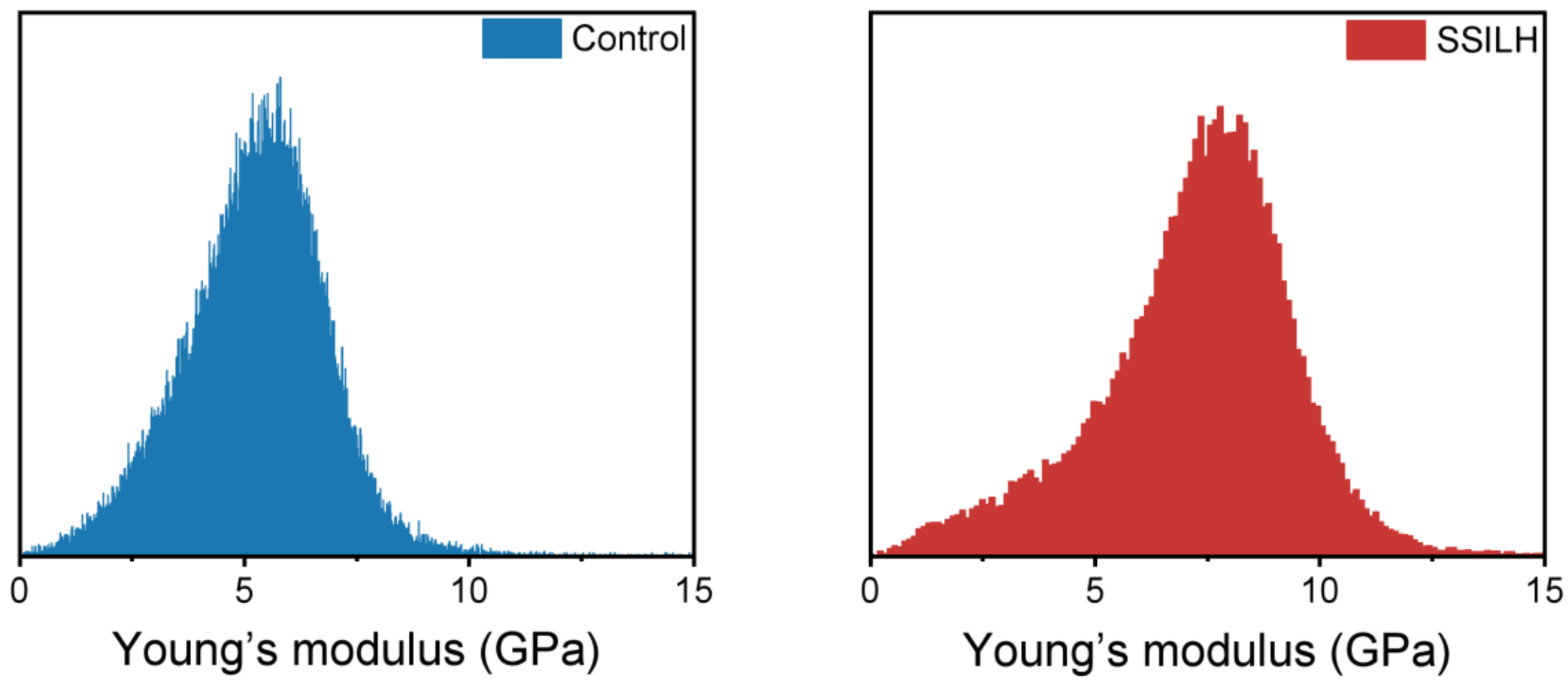

Figure S8 |

The distribution of Young's modulus for the surface of control and SSILH perovskite.

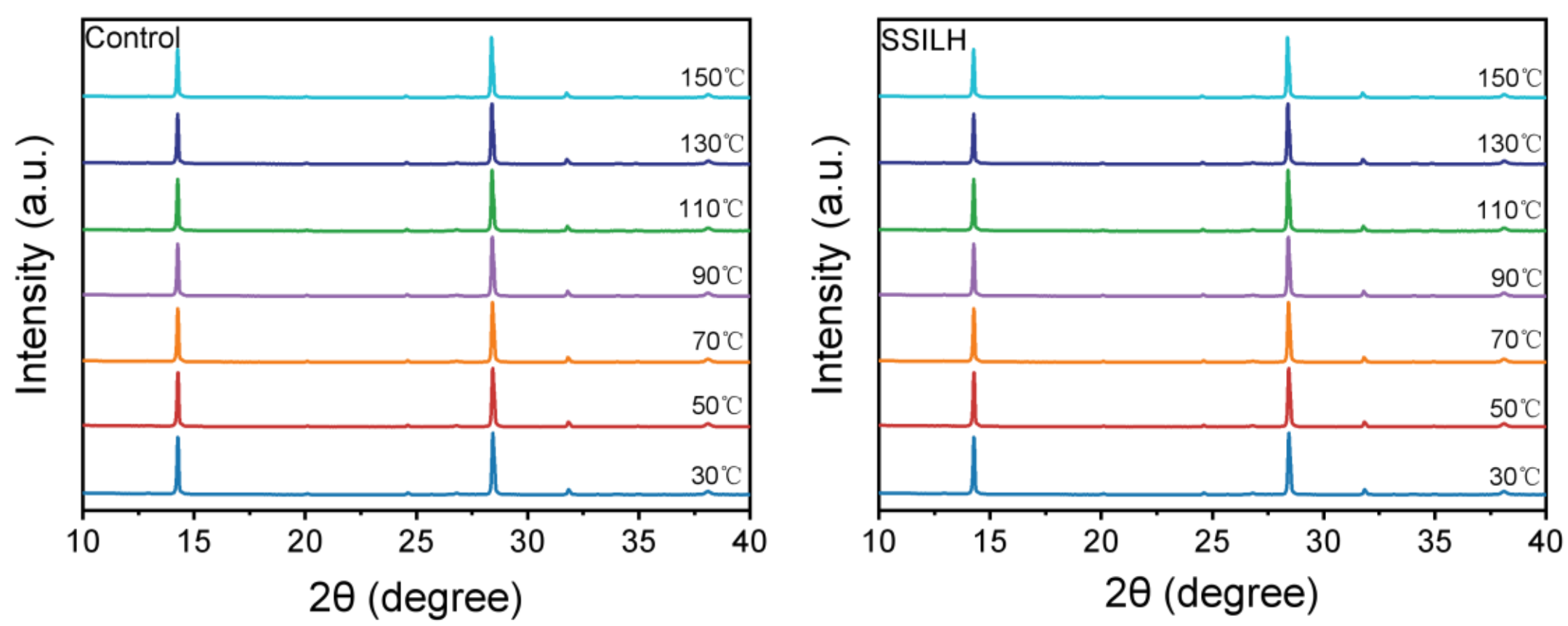

Figure S9 | Crystal structures analysis of perovskite at different temperatures.

In-situ X-ray diffraction spectra of the control and SSILH perovskite films at different temperatures.

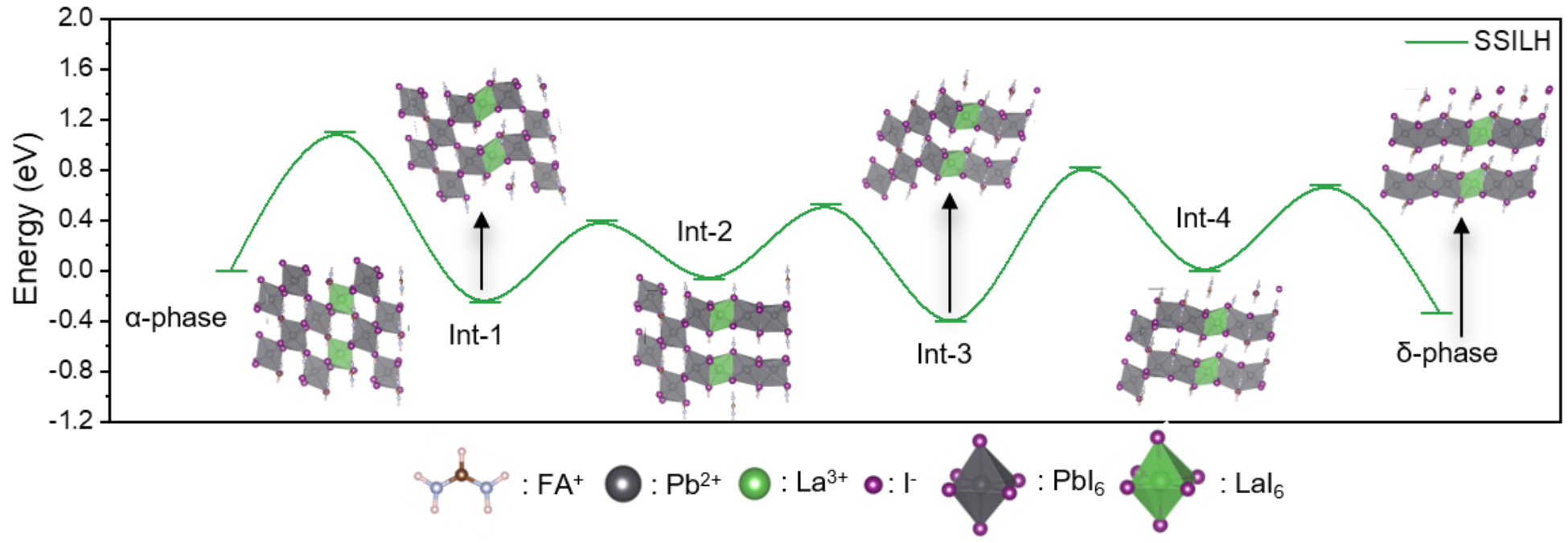

Figure S10 | Calculation of the SSILH $FAPbI_3$ phase transition process.

The phase transformation process from left to right is black-phase (α-phase), the first interruption of La–I bonds (Int-1), further breaking of Pb–I bonds (Int-2, Int-3, and Int-4), and yellow-phase (δ-phase).

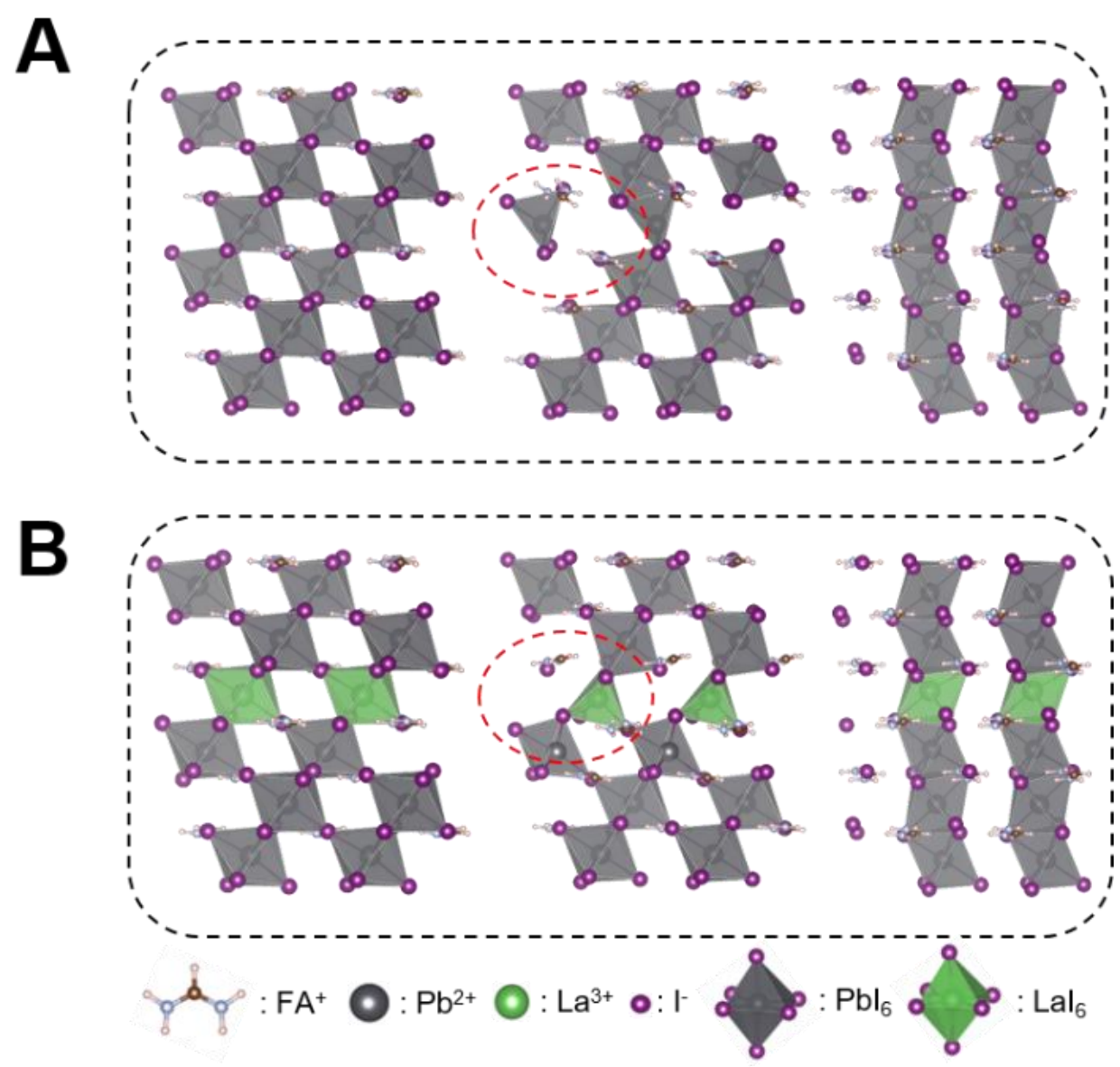

Figure S11 | Progression of the structural transition from the corner-sharing cubic structure to the hexagonal face-sharing structure through breaking the B-X bonds.

The phase transition in $FAPbI_3$ perovskite starts from breaking the Pb-I bonds and forming a one-dimensional PbI_4 tetrahedral chain at the transition state. The (A) control and (B) SSILH $FAPbI_3$.

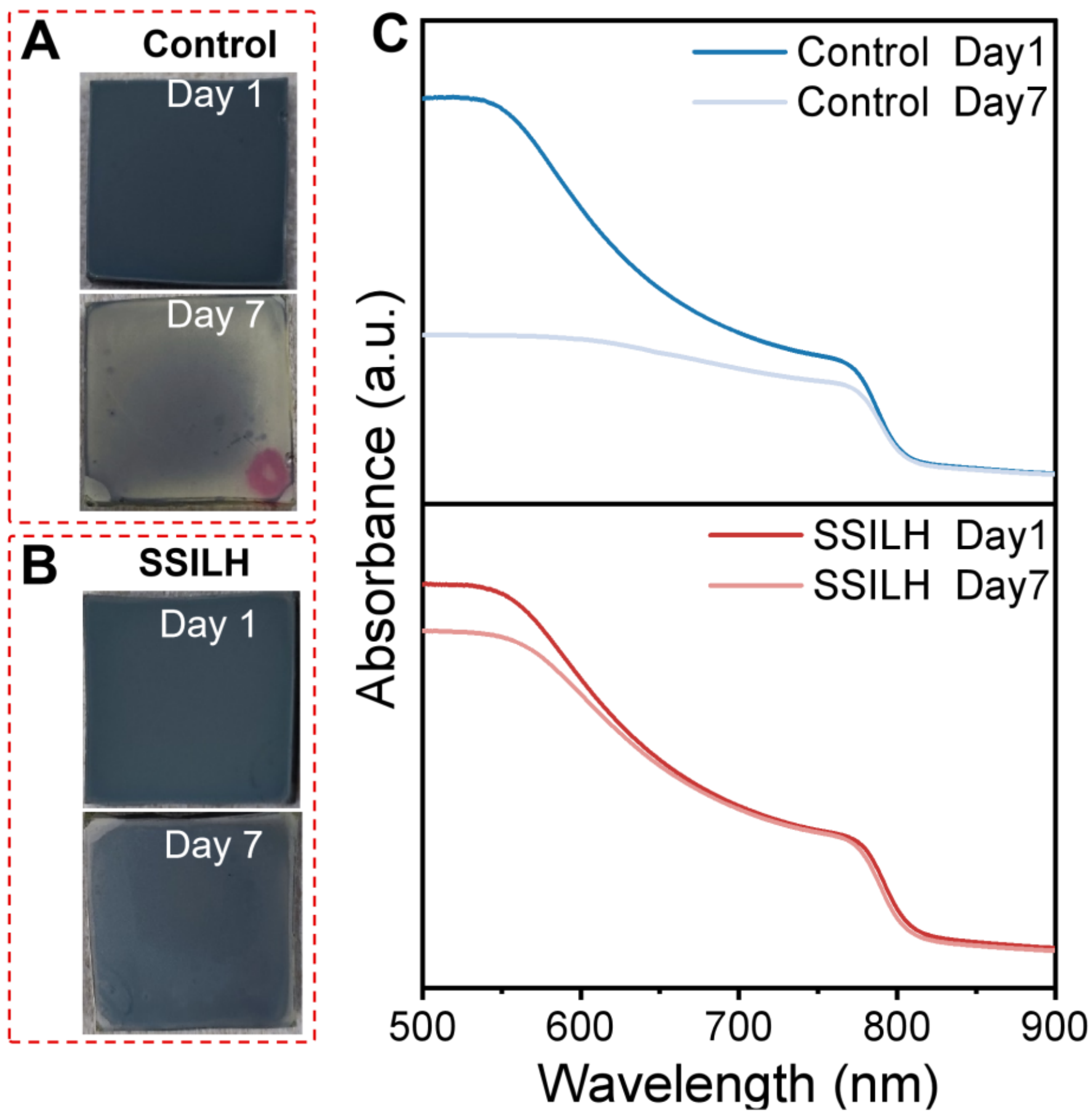

Figure S12 | Stability test of the control and SSILH perovskite films.

The (**A**) control and (**B**) SSILH perovskite films before and after exposure for 7 days in ambient air (RH≈50%). (**C**) The UV-vis absorption spectrum of the control and SSILH perovskite films before and after exposure for 7 days in ambient air (RH≈50%).

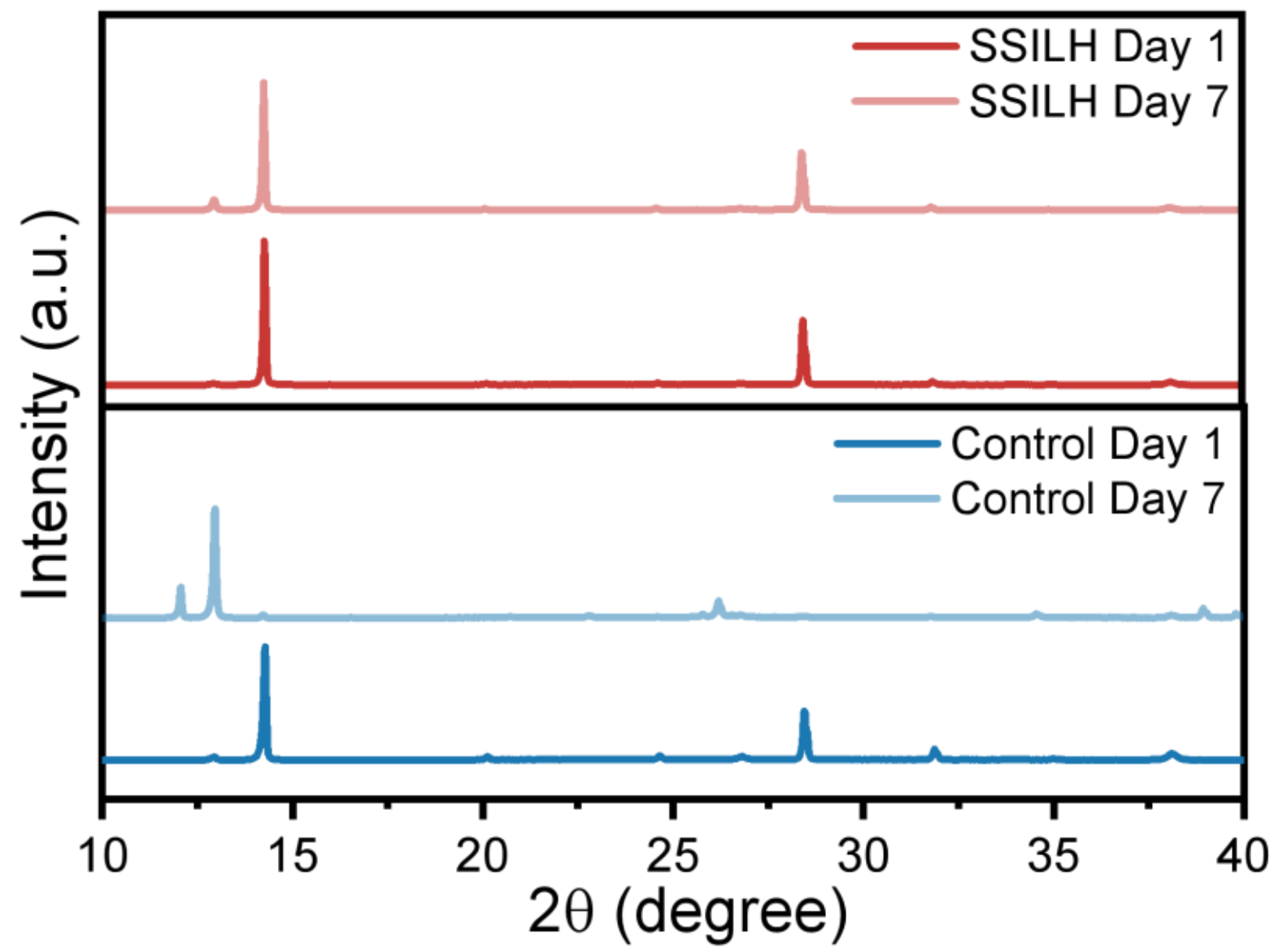

Figure S13 | Analysis of humidity stability of the perovskite films.

XRD patterns of the control and SSILH perovskite films before and after exposure for 7 days in ambient air (RH≈50%).

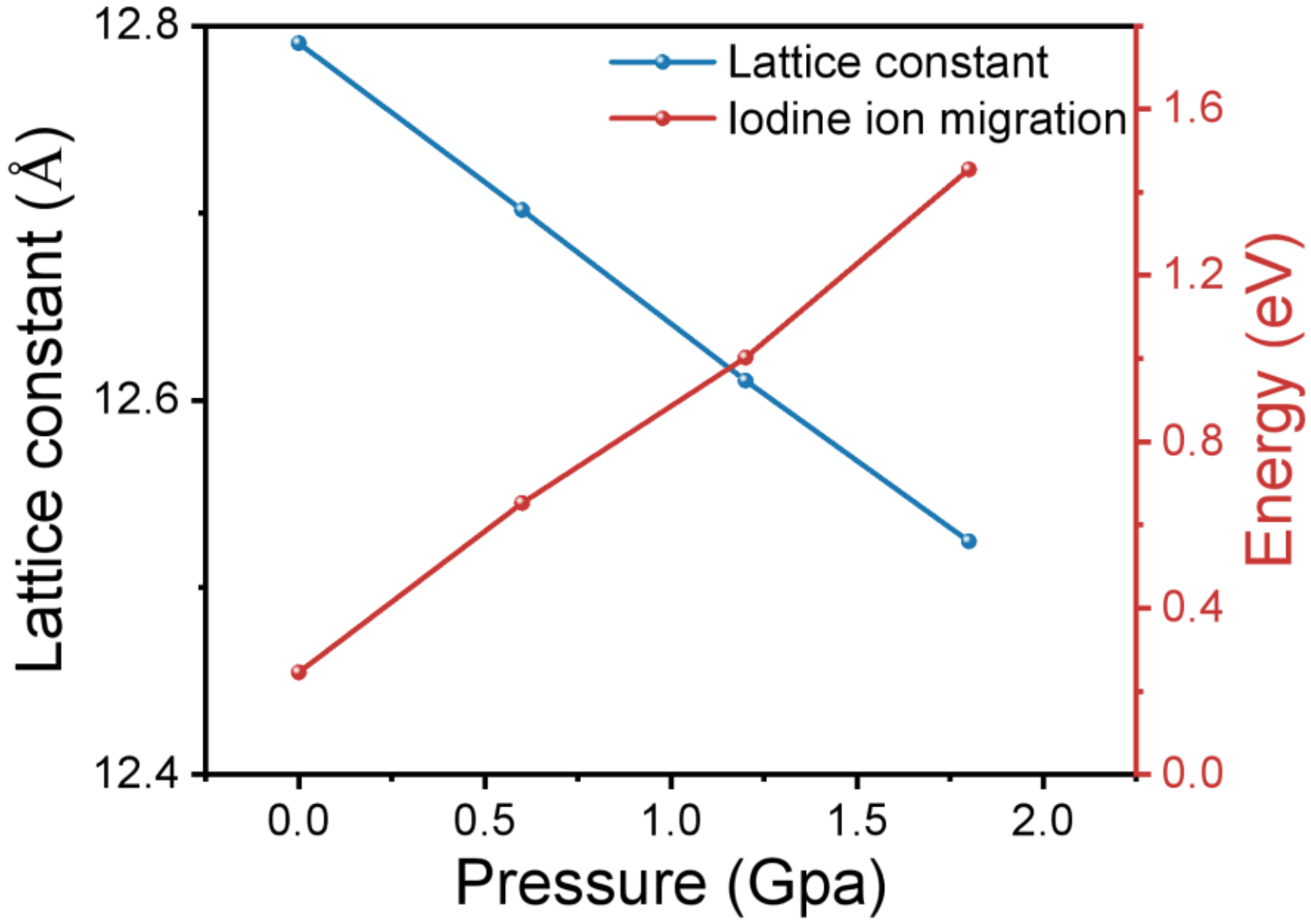

Figure S14 | Calculation of the average lattice constant and iodide ion migration barriers under different pressures.

The calculation method of the iodide ion migration energy barrier is as follows: An iodide vacancy (V_I) was initially set in two neighboring positions of a BX_6 octahedron, corresponding to the defects in the initial and final structure, respectively. Initial and final structures were first optimized, then a linear interpolation consisting of 6 grid points between them were employed to define the migration process. The energy profiles were examined using climbing image nudged elastic band method (cl-NEB) and Limited-memory Broyden-Fletcher-Goldfarb-Shanno (LBFGS) energy minimization methods.

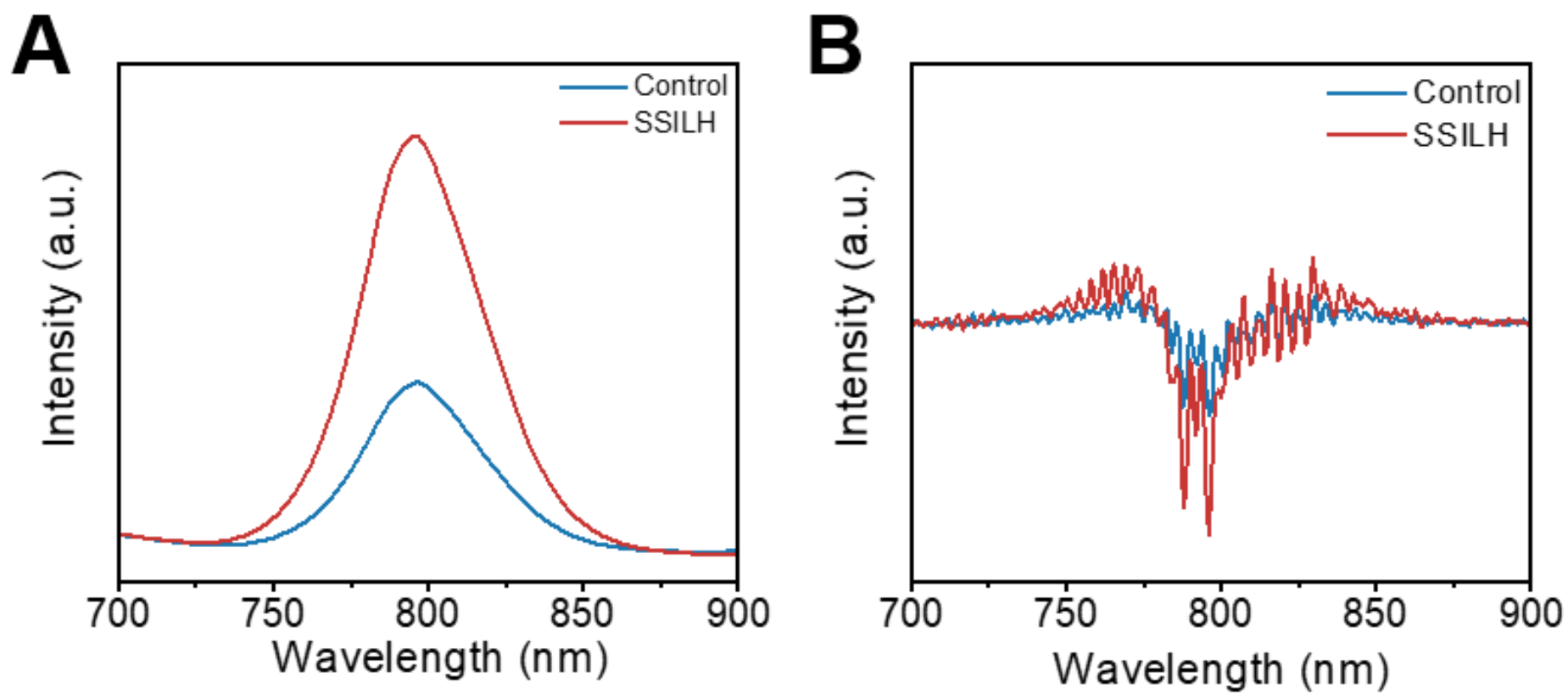

Figure S15 |

(A) The steady-state PL spectrum of the control and SSILH perovskite films. (B) The second derivative graphs of the control and the SSILH perovskite films.

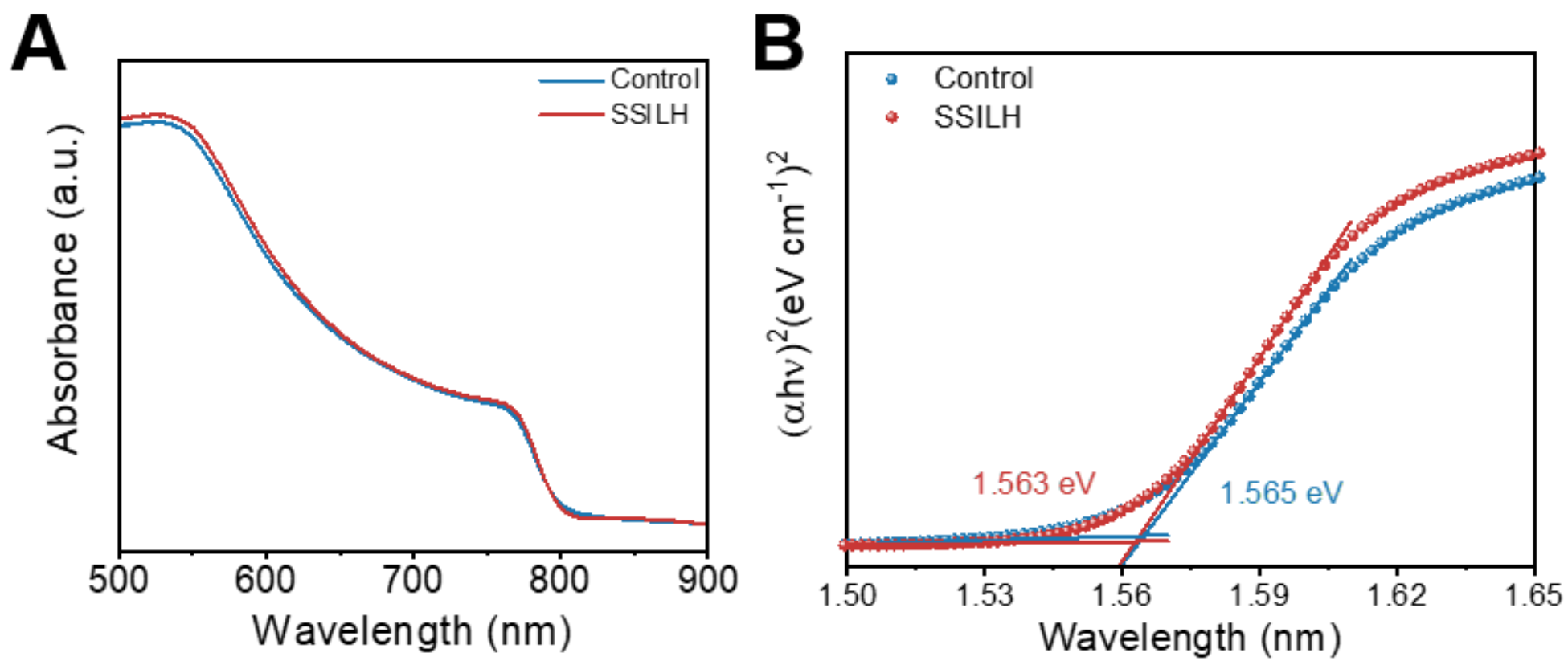

Figure S16 |

(A) The UV-vis absorption spectrum of the control and SSILH perovskite films. (B) The Tauc Plots of the control and SSILH perovskite films.

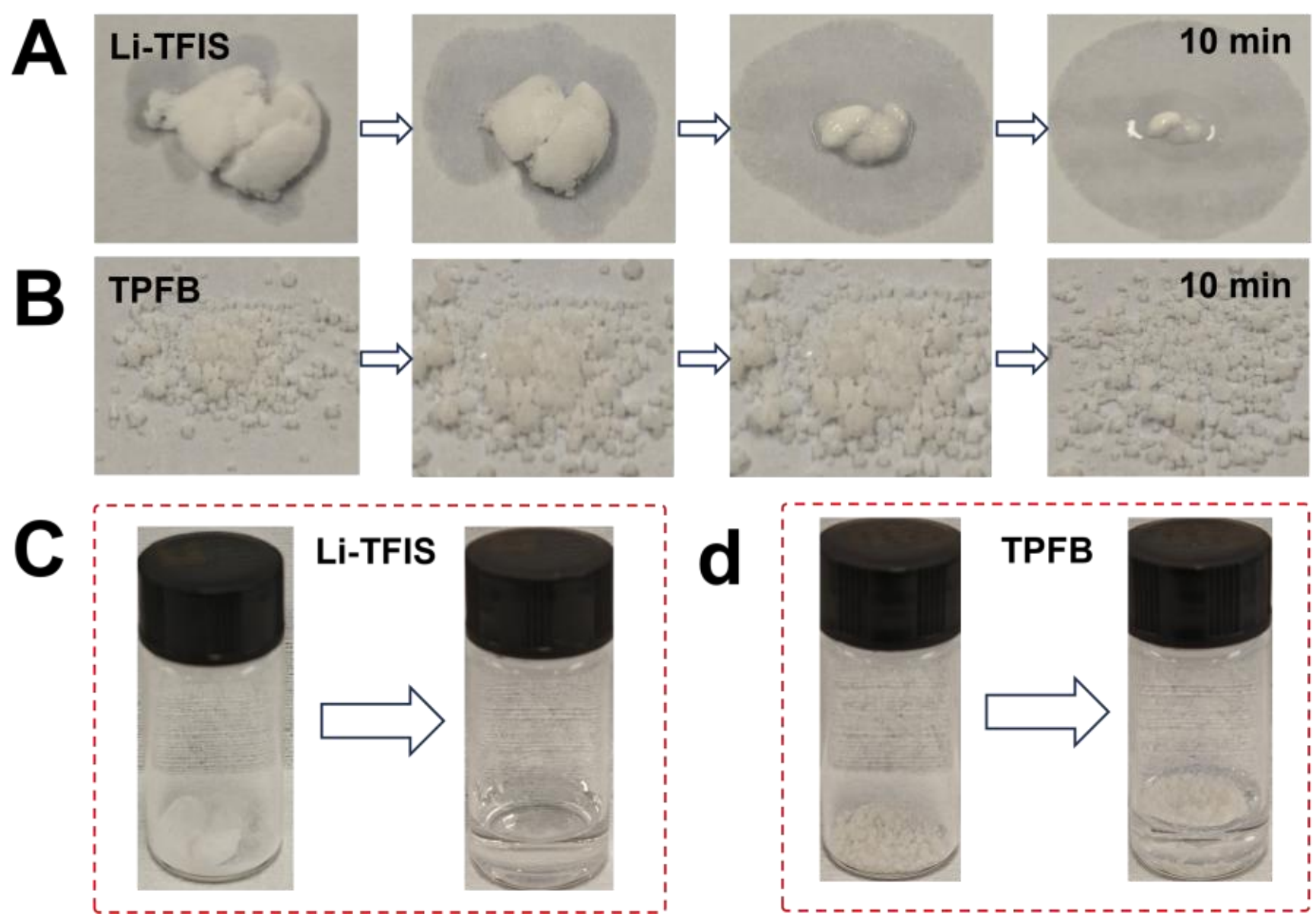

Figure S17 | Comparison of hygroscopicity between Li-TFSI and TPFB.

The (**A**) Li-TFSI and (**B**) TPFB powder were exposed for 10 min in ambient air (RH=40~50%). The (**C**) Li-TFSI and (**D**) TPFB were added to the water. We observed that the Li-TFSI completely dissolved in water, while the TPFB was almost insoluble in water.

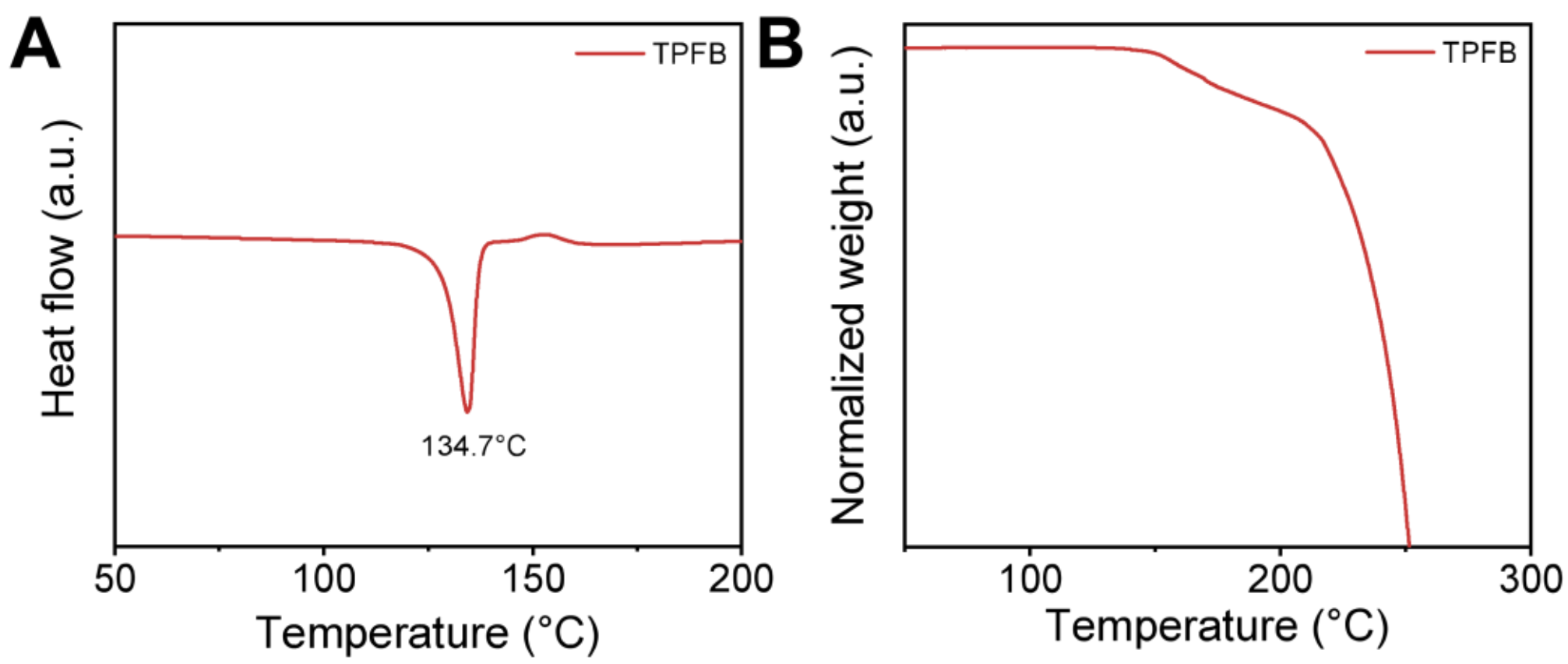

Figure S18 | The thermodynamic properties of TPFB.

(**A**) The differential scanning calorimetry and (**B**) thermogravimetric analysis of TPFB powder. The glass transition point of TPFB is approximately 134.7℃.

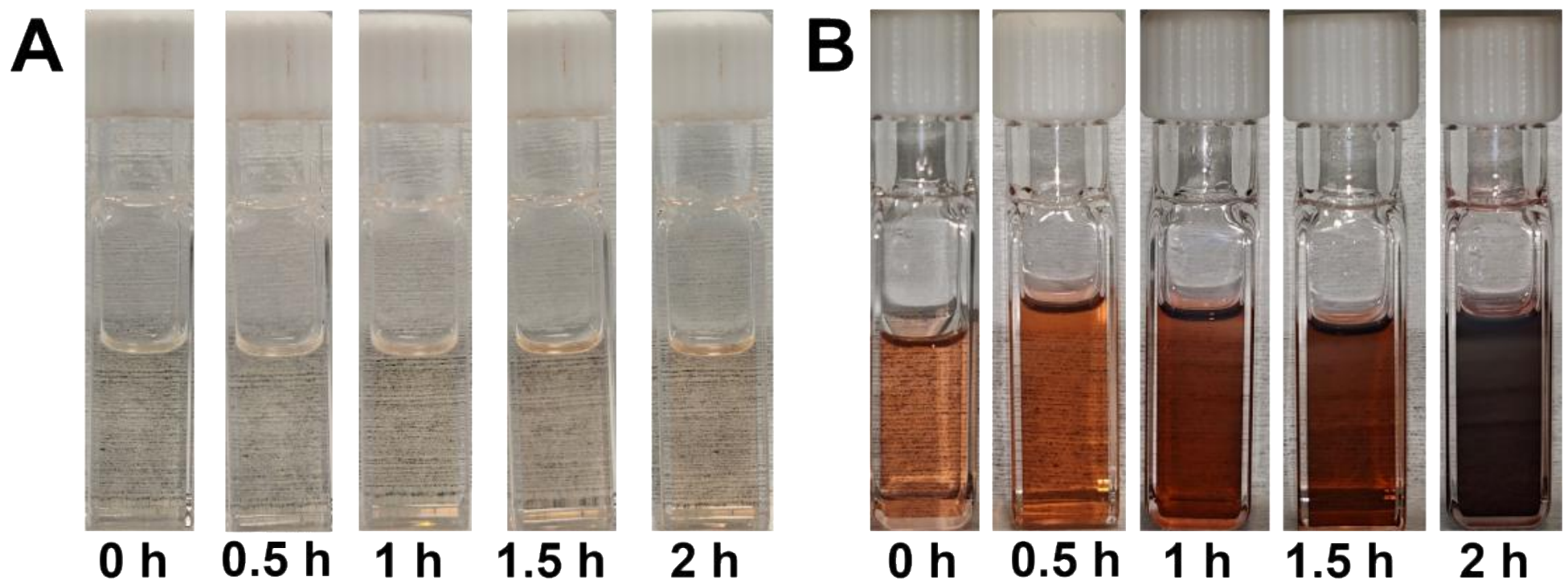

Figure S19 |

Schematic diagram of Spiro-OMeTAD CB solution with different dopants aging over time at room temperature without exposure to O_2 or air. (**A**) *t*BP/Li-TFSI and (**B**)TPFB.

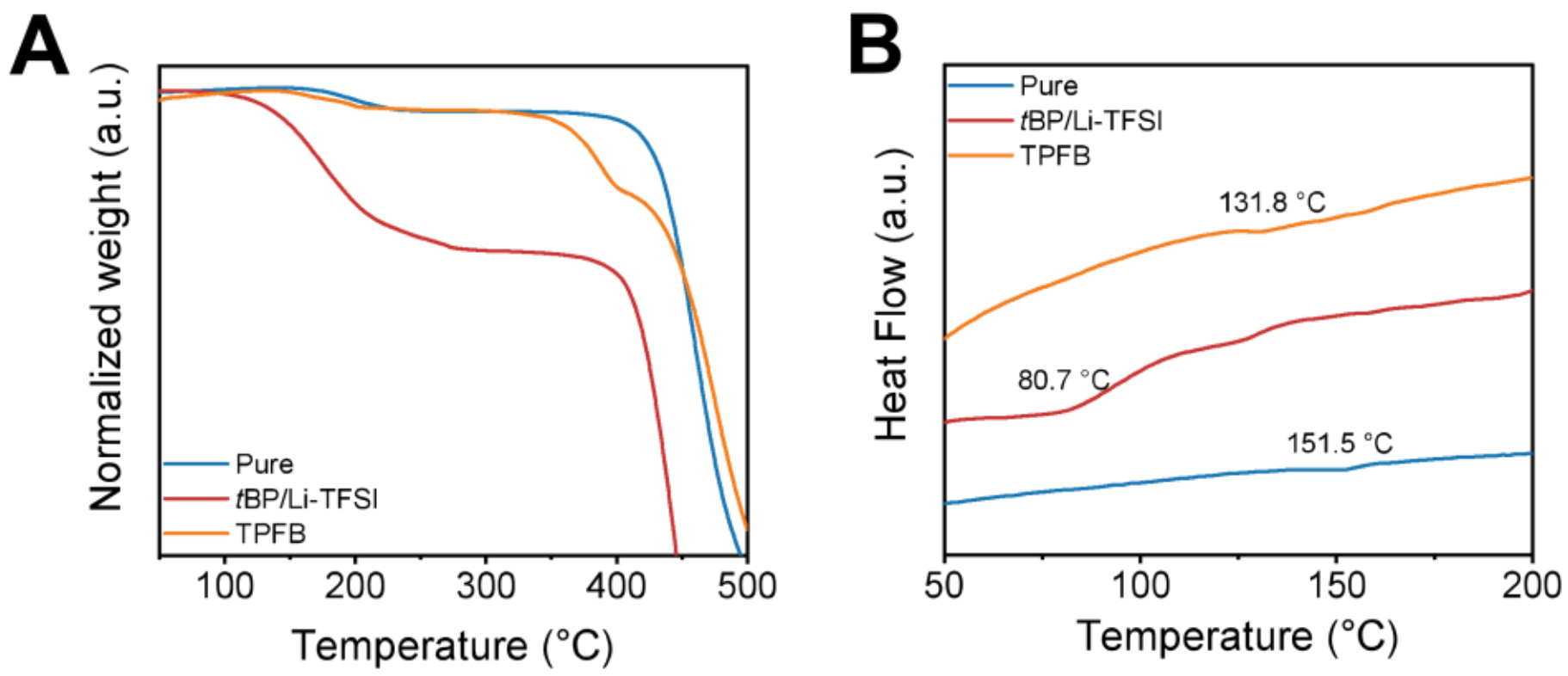

Figure S20 | Thermodynamic properties of Spiro-OMeTAD powders based on different dopants.

(**A**) The thermogravimetric analysis and (**B**) differential scanning calorimetry of Pure, *t*BP/Li-TFSI and TPFB-doped Spiro-OMeTAD powder. The pure Spiro-OMeTAD has a glass transition temperature of 151.5℃, while after adding the *t*BP/Li-TFSI dopant, its glass transition point obviously decreases to 80.7℃, which is consistent with previous reports [6,7]. In comparison, the TPFB-doped Spiro-OMeTAD possess a much higher glass transition point of 131.8℃, illustrating that the TPFB resulted in a better thermal stability of HTL than the conventional tBP/Li-TFSI dopant.

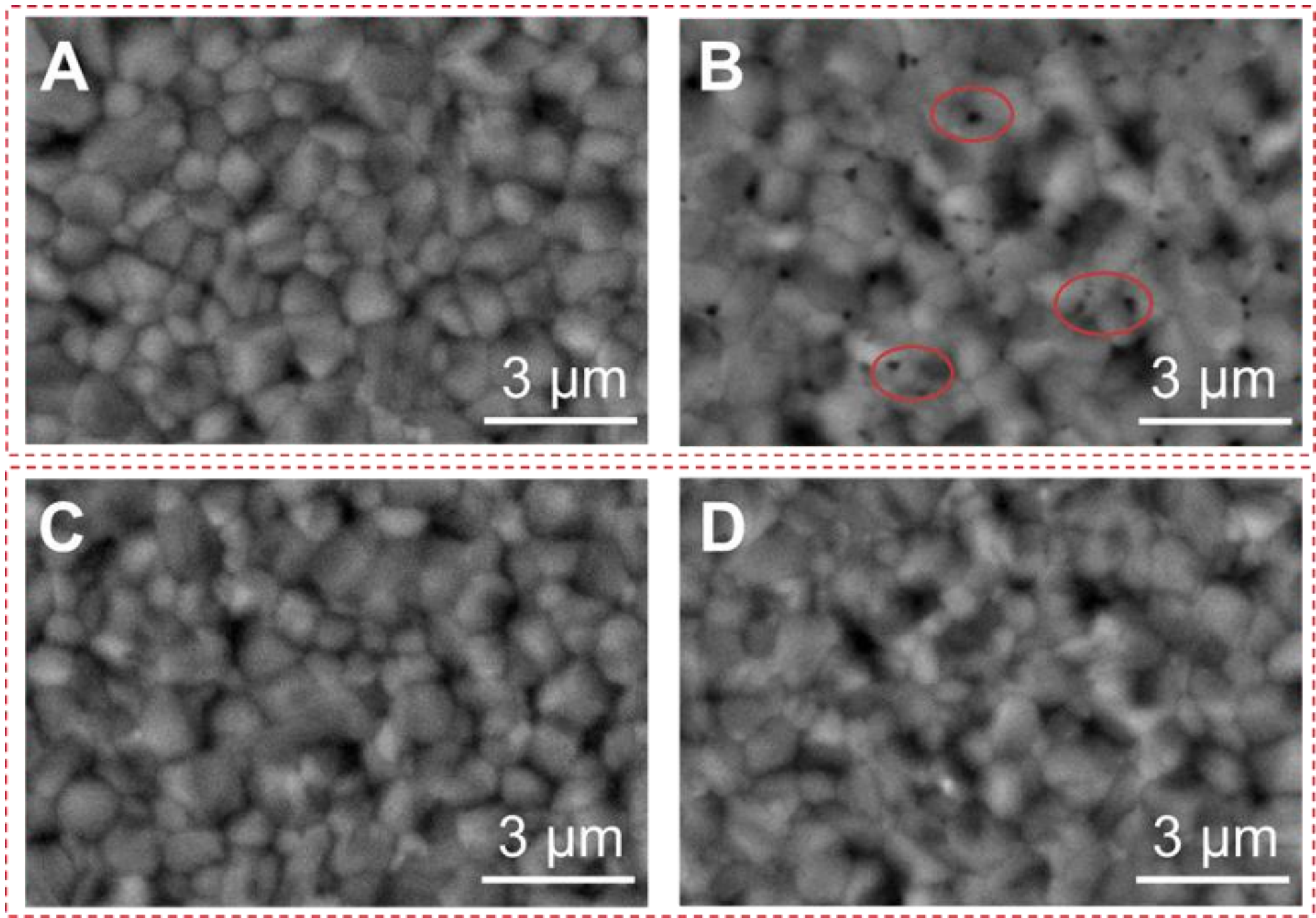

Figure S21 | SEM images of the perovskite /Spiro-OMeTAD films.

Li-TFSI/tBP doped Spiro-OMeTAD (A) before and (B) after 85°C treatments. TPFB-doped Spiro-OMeTAD (C) before and (D) after 85°C treatments. It was observed that the Li-TFSI/tBP doped Spiro-OMeTAD developed numerous pinholes after thermal treatment at 85°C. In comparison, the TPFB-doped Spiro-OMeTAD film maintains excellent morphological stability without any visible pinholes in the same condition.

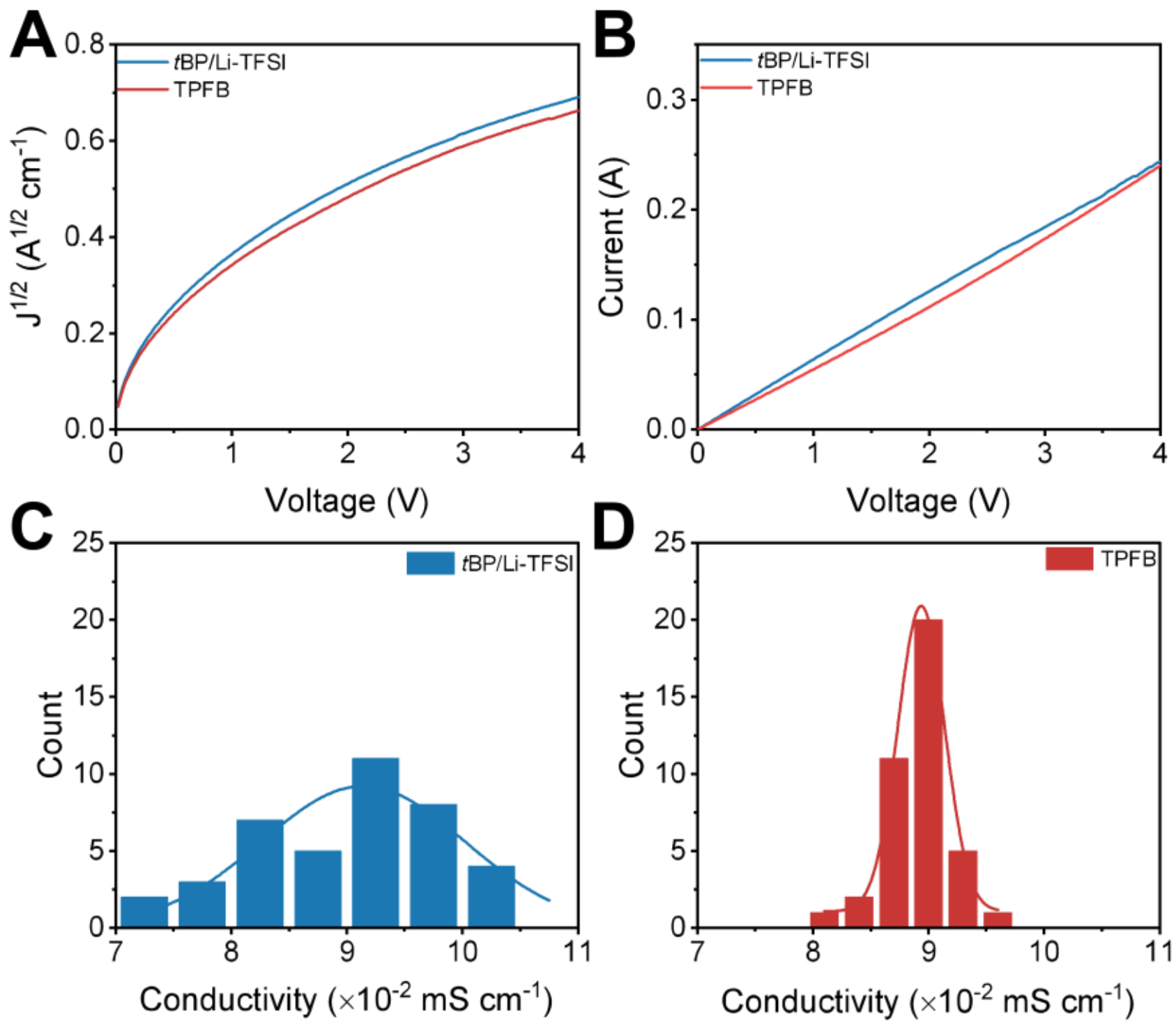

Figure S22 | Comparable hole-mobility and conductivity.

(**A**) A typical hole mobility of *t*BP/Li-TFSI and TPFB doped Spiro-OMeTAD films. (**B**) A typical conductivity of *t*BP/Li-TFSI and TPFB doped Spiro-OMeTAD films. The statistics distribution of conductivity of (**C**) *t*BP/Li-TFSI and (**D**) TPFB-doped Spiro-OMeTAD. The dispersed conductivity distribution of *t*BP/Li-TFSI doped Spiro-OMeTAD is due to the oxidation process is seriously affected by the external environment, while the concentrated conductivity distribution of TPFB-doped Spiro-OMeTAD should be due to the more stable oxidation state (without needing oxygen-assisted oxidation).

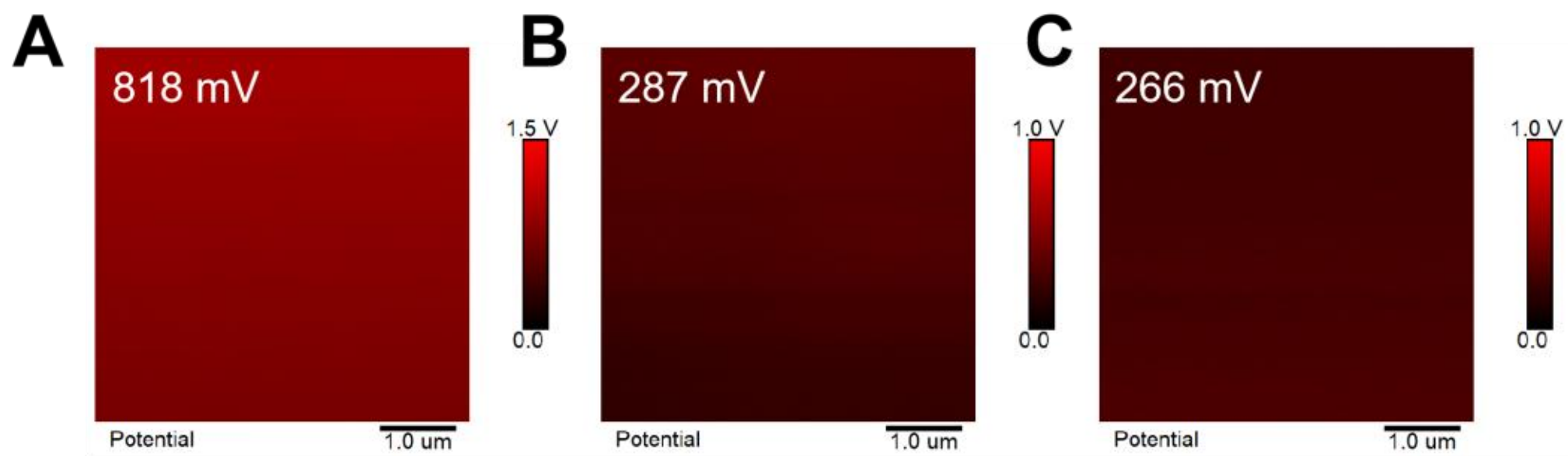

Figure S23 | Surface potential measurements of Spiro-OMeTAD films based on different dopants.

KPFM images and corresponding surface potential of (**A**) Pure Spiro-OMeTAD (The average electric potential is 818 mV and the W_F is 4.282 eV), (**B**) *t*BP/Li-TFSI doped Spiro-OMeTAD (The average electric potential is 287 mV and the W_F is 4.813 eV), and (**C**) TPFB-doped Spiro-OMeTAD (The average electric potential is 266 mV and the W_F is 4.834 eV). The work function of a gold sample as a reference is approximately 5.1 eV.

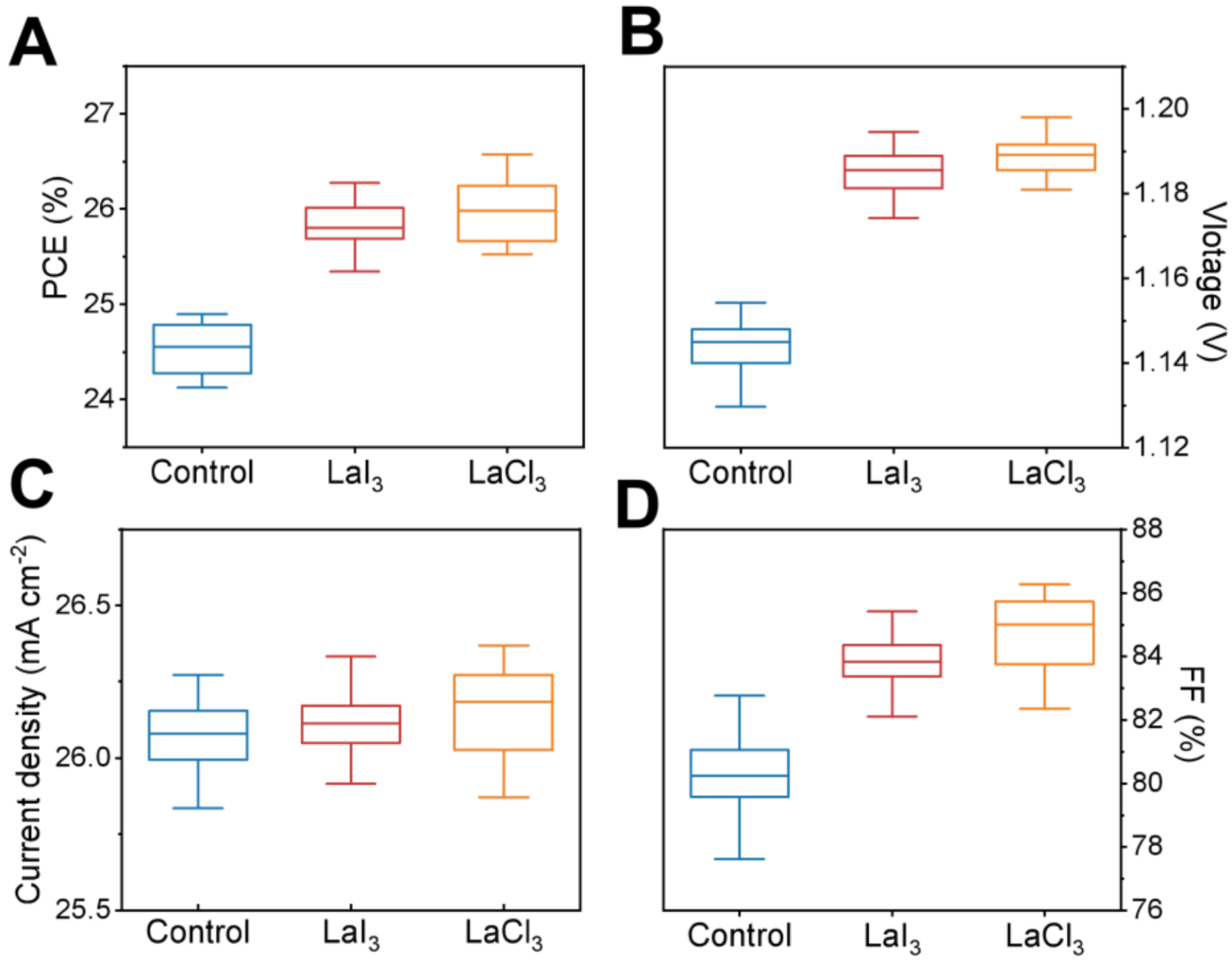

Figure S24 | The statistical distribution for 20 devices fabricated with doping different kinds of Lanthanum halide in the perovskite precursor.

(**A**) PCE, (**B**) V_{OC}, (**C**) J_{SC}, and (**D**) FF. Center line: median, box limits: 25th (the lower) and 75th (the upper) percentile.

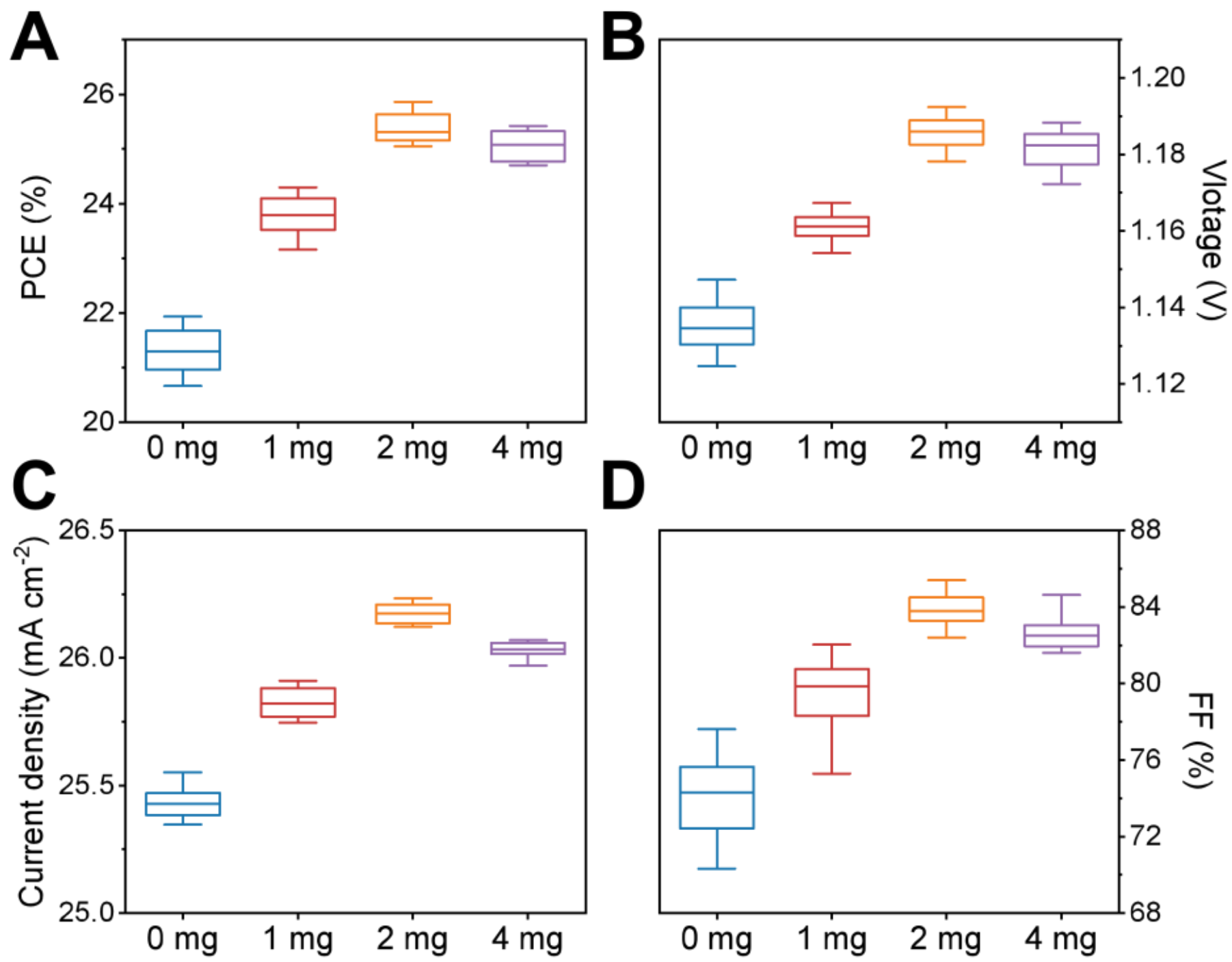

Figure S25 | Photovoltaic performances of the SSILH devices based on different concentrations of TPFB-doped Spiro-OMeTAD.

Statistical photovoltaic parameters of (**A**) PCE, (**B**) V_{OC}, (**C**) J_{SC}, and (**D**) FF. 20 devices were collected from the different batches. Center line: median, box limits: 25th (the lower) and 75th (the upper) percentile.

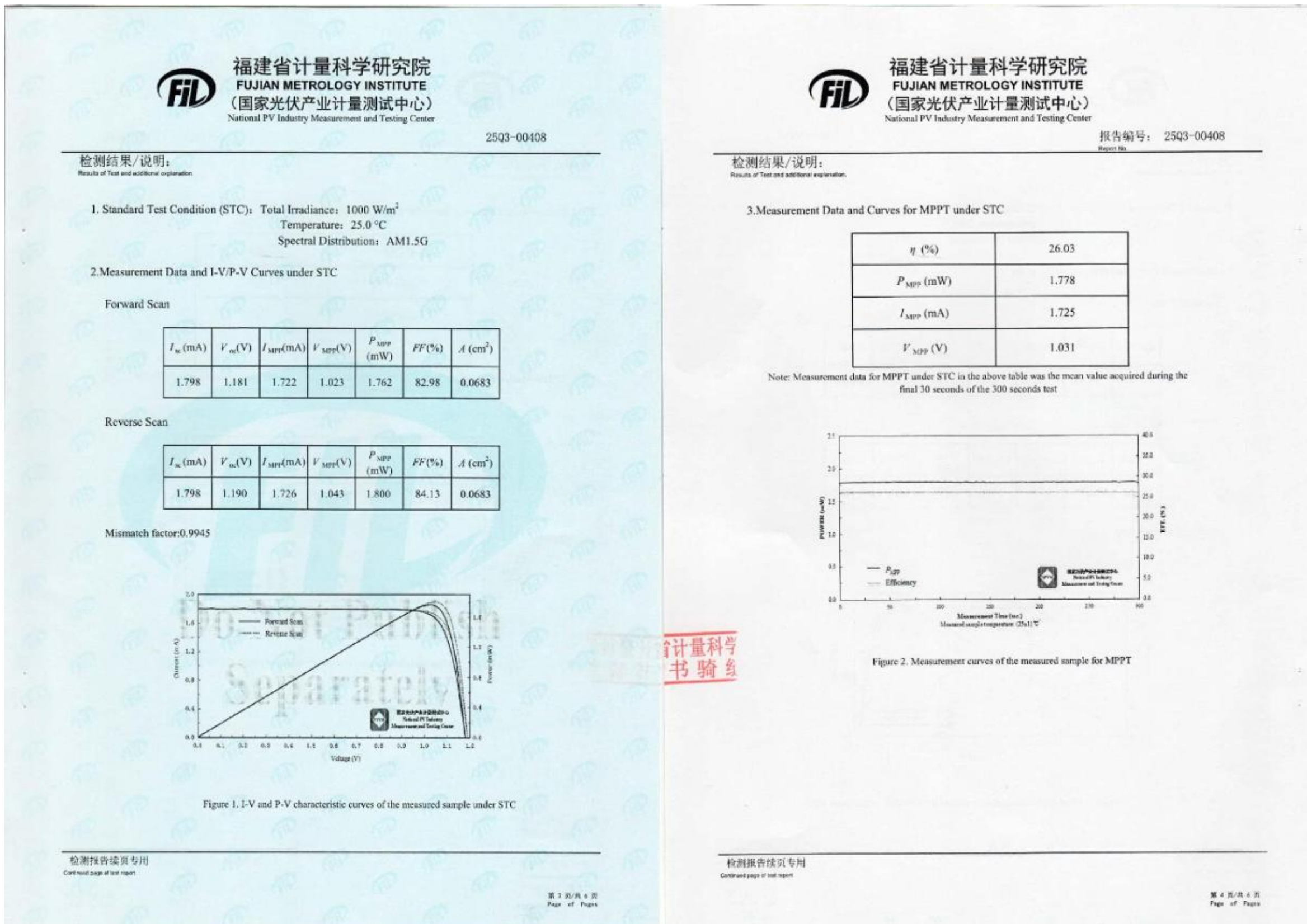
福建省计量科学研究院
FUJIAN METROLOGY INSTITUTE
（国家光伏产业计量测试中心）
National PV Industry Measurement and Testing Center

25Q3-00408

检测结果/说明：
Results of Test and additional explanation

1. Standard Test Condition (STC)： Total Irradiance： 1000 W/m²
Temperature： 25.0 °C
Spectral Distribution： AM1.5G

2.Measurement Data and I-V/P-V Curves under STC

Forward Scan

I_{sc} (mA)	V_{oc}(V)	I_{MPP}(mA)	V_{MPP}(V)	P_{MPP} (mW)	FF(%)	A (cm²)
1.798	1.181	1.722	1.023	1.762	82.98	0.0683

Reverse Scan

I_{sc} (mA)	V_{oc}(V)	I_{MPP}(mA)	V_{MPP}(V)	P_{MPP} (mW)	FF(%)	A (cm²)
1.798	1.190	1.726	1.043	1.800	84.13	0.0683

Mismatch factor:0.9945

Figure 1. I-V and P-V characteristic curves of the measured sample under STC

检测报告续页专用
Continued page of test report

第 3 页/共 6 页
Page of Pages

福建省计量科学研究院
FUJIAN METROLOGY INSTITUTE
（国家光伏产业计量测试中心）
National PV Industry Measurement and Testing Center

报告编号： 25Q3-00408
Report No.

检测结果/说明：
Results of Test and additional explanation.

3.Measurement Data and Curves for MPPT under STC

η (%)	26.03
P_{MPP} (mW)	1.778
I_{MPP} (mA)	1.725
V_{MPP} (V)	1.031

Note: Measurement data for MPPT under STC in the above table was the mean value acquired during the final 30 seconds of the 300 seconds test

Figure 2. Measurement curves of the measured sample for MPPT

检测报告续页专用
Continued page of test report

第 4 页/共 6 页
Page of Pages

Figure S26 | Third-party certification.

The certificate of one SSILH device based on TPFB-doped Spiro-OMeTAD (*J*-*V* characterization). The report is issued by NPVM (National PV Industry Measurement and Testing Center). The report includes the optical aperture area, *J*-*V*, and MPP tracking measurement.

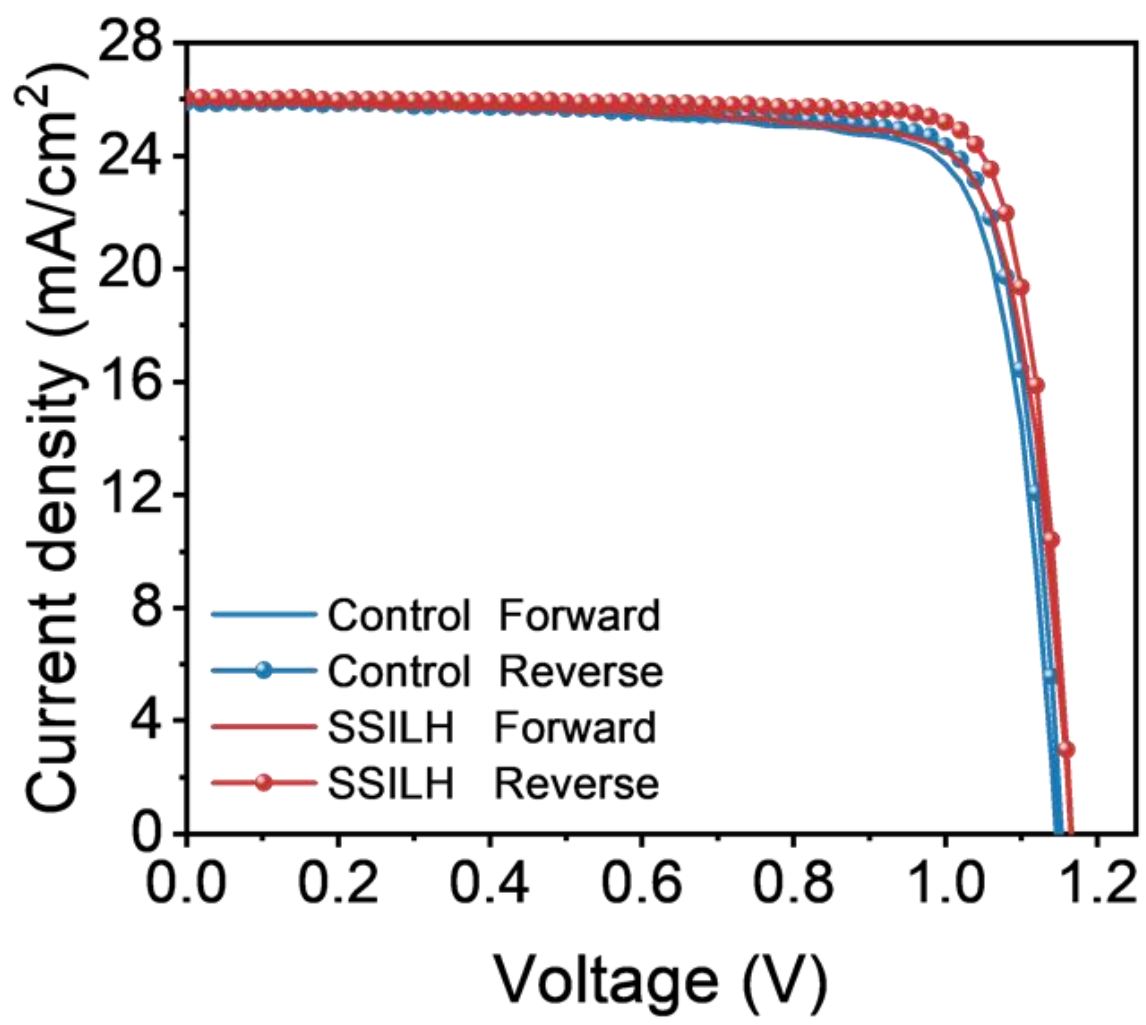

Figure S27 | J-V curves of the control and SSILH devices with a p-i-n structure.

The power conversion efficiency (PCE) of the control device is 24.32% with a short-circuit current density (J_{SC}) of 25.98 mA cm^{-2}, an open-circuit voltage (V_{OC}) is 1.149 V and a filling factor (FF) of 81.48% under reverse scanning. In comparison, the SSILH device yielded a PCE of 25.27% with a J_{SC} of 26.04 mA cm^{-2}, a V_{OC} of 1.162 V and a FF of 83.50% under reverse scanning.

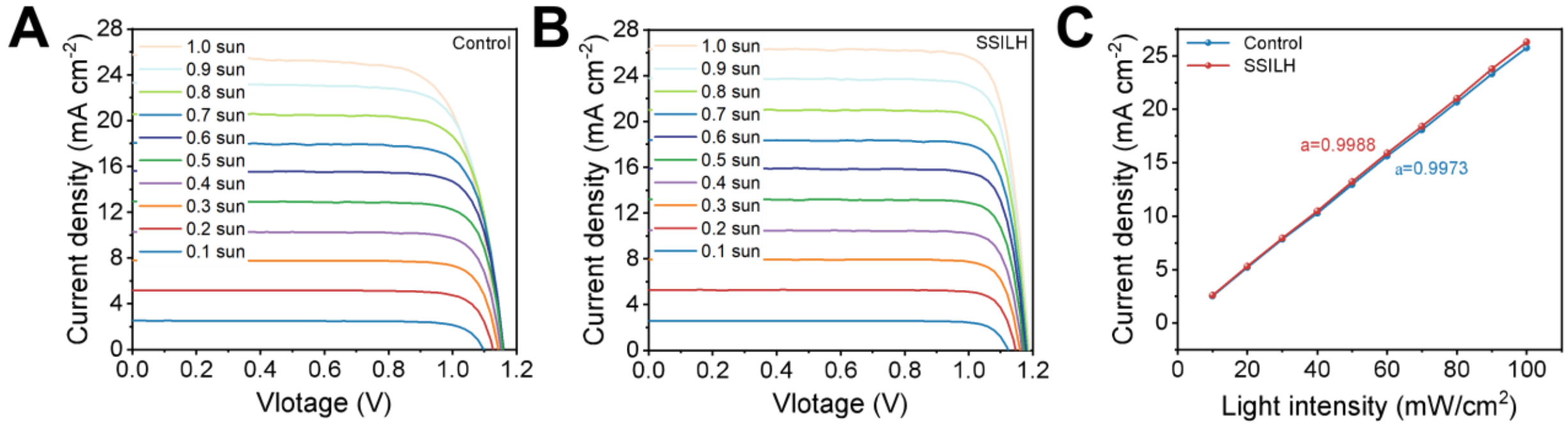

Figure S28 | The ideal factor of different solar cells.

The response of solar cells under different light intensities for the (**A**) control and (**B**) SSILH devices. (**C**) Light intensity dependence of J_{SC} for the control and SSILH devices. The ideality factor was calculated from the slope of the linear fit of the semilogarithmic plot.

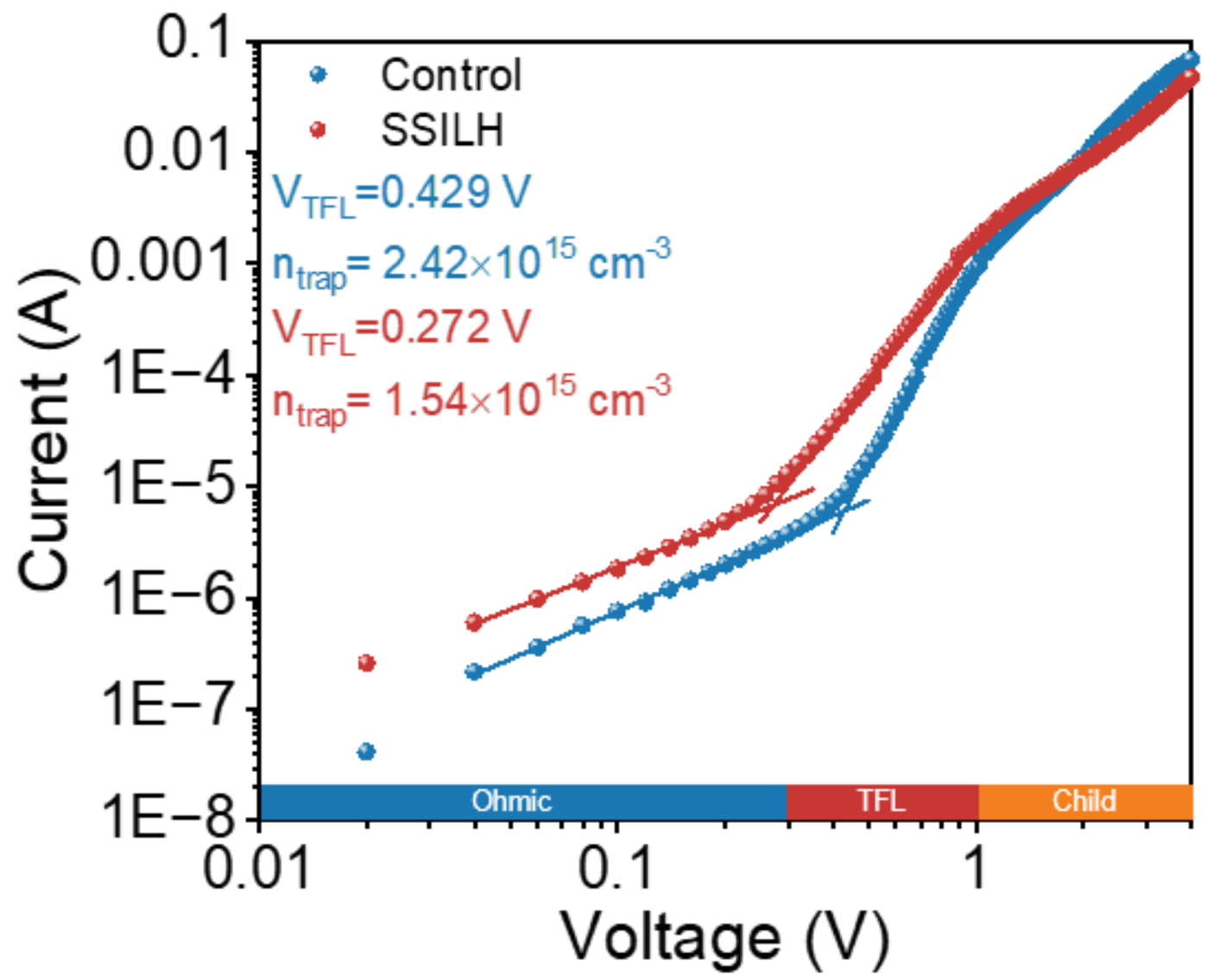

Figure S29 | Trap density characterization.

Dark current-voltage measurements of the hole-only devices (FTO/PEDOT:PSS/Perovskite/Spiro-OMeTAD/Au). V_{TFL} is the trap-filled limit voltage. n_{trap} is the trap density.

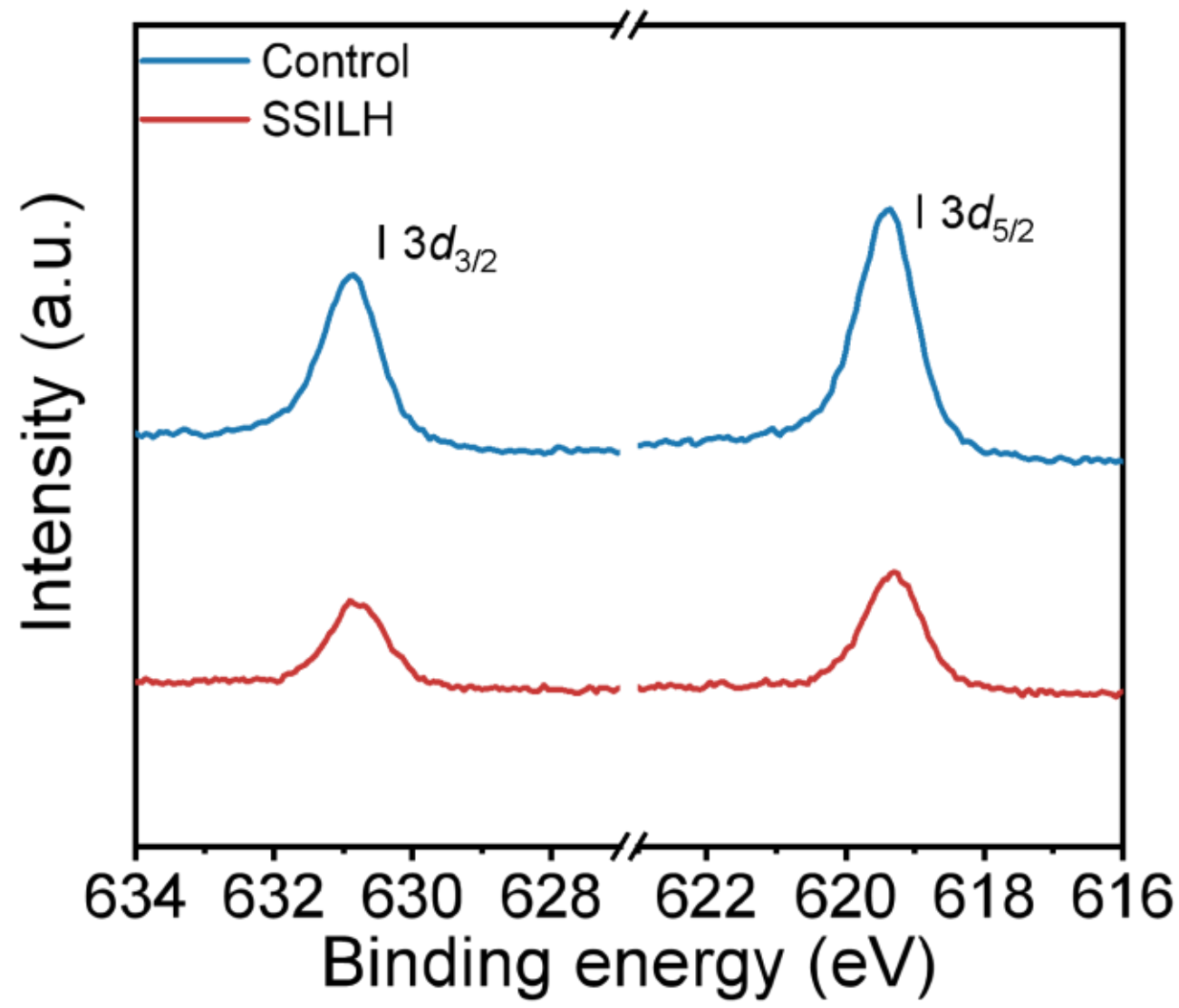

Figure S30 | XPS measurement of gold electrode surface after stability test.

XPS signal of the I element on the gold electrode surface of the control and SSILH devices after long-term operational stability test.

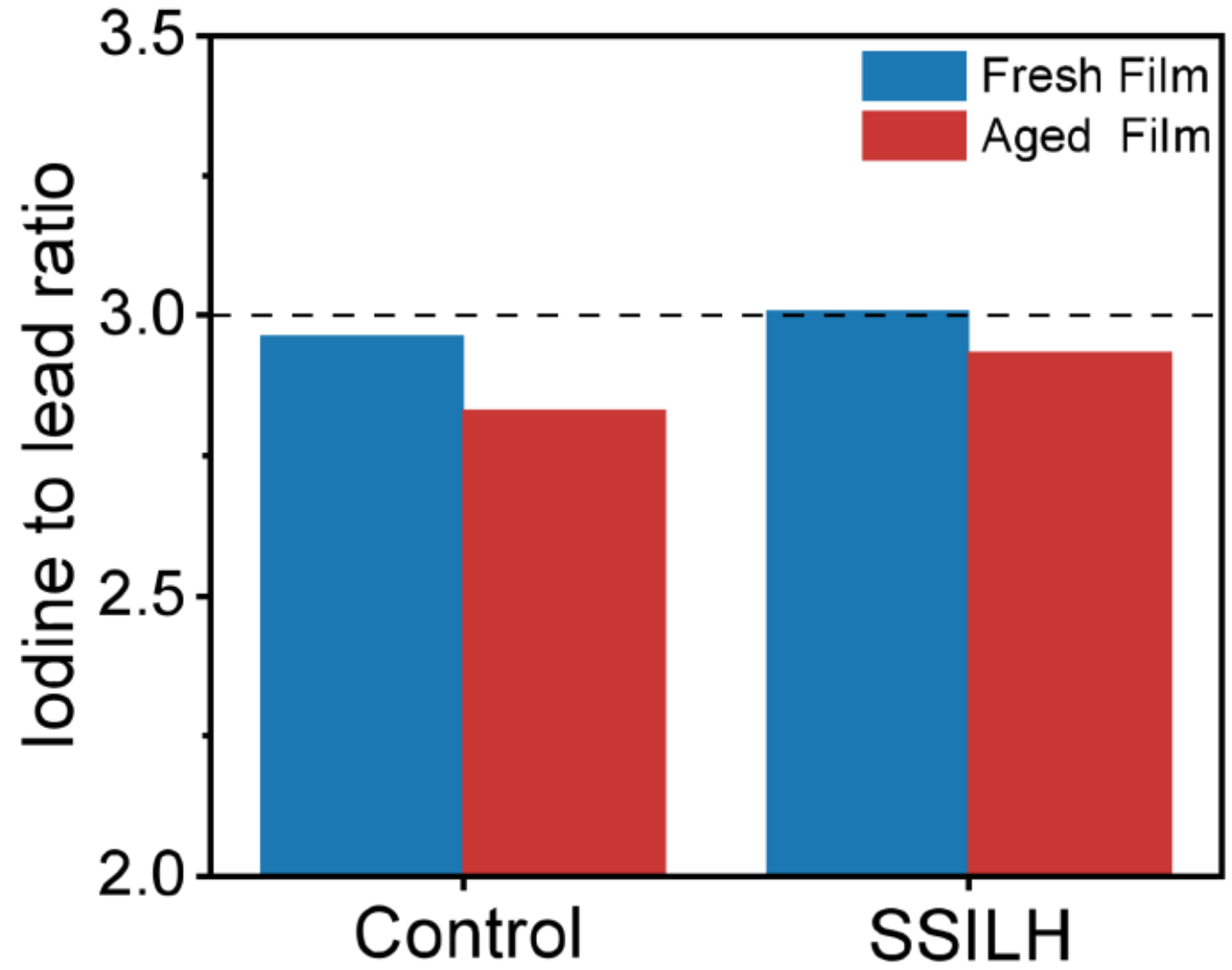

Figure S31 | EDS measurements of the control and SSILH perovskite films.

Iodide-to-lead ratio of the control and SSILH perovskite films before (fresh device) and after heating (aged device) at 85℃ for 21 days.

Table S1. Crystal structure parameters of $FAPbI_3$ (control) and La-doped $FAPbI_3$ (SSILH) calculated by DFT. We constructed a 2×2×2 $FAPbI_3$ supercell.

Name	a (Å)	b (Å)	c (Å)	V ($Å^3$)
Control	12.828	12.475	12.630	2021.170
SSILH	12.805	12.459	12.614	2012.406
Rate of change (%)	0.179	0.128	0.127	0.433

Table S2. The relative element content in SSILH perovskite films.

Element	Mass (%)	Atom (%)
C	7.10	46.67
N	0.69	3.88
Pb	32.66	12.45
I	58.29	36.28
La	1.26	0.72
Total	100.00	100.00

Table S3. The Young's modulus (Er) and hardness (H) of the control and SSILH perovskite films.

Samples	Er (GPa)	H (GPa)
Control	27.96	0.70
SSILH	35.71	0.91

Table S4. The lattice constant changes of the control and SSILH perovskite films before and after heating.

Samples	2θ (º)	Lattice constant (Å)	Thermal expansion rate (%)	Coefficient of thermal expansion ($\times 10^{-6}$ 1/℃)
Control (30ºC)	14.02	6.312	0.21	17.50
Control (150ºC)	13.99	6.325		
SSILH (30ºC)	14.03	6.307	0.08	6.67
SSILH (150ºC)	14.02	6.312		

Table S5. Photovoltaic parameters of PSCs measured at simulated AM 1.5G solar irradiation.

Samples	V_{OC} (V)	J_{SC} (mA cm^{-2})	FF (%)	PCE (%)	HI
Control-Forward	1.158	26.07	79.08	23.87	0.0379
Control-Reverse	1.164	26.02	81.93	24.81	
SSILH-Forward	1.197	26.09	83.78	26.16	0.0143
SSILH-Reverse	1.196	26.08	85.10	26.54	

Table S6. Photovoltaic parameters of n-i-p PSCs measured at simulated AM 1.5G solar irradiation.

Dopant of HTL	PCE	quasi-steady PCE	Ref.
Li-TFSI + *t*BP	25.73%*	-	*Nature* **616**, 724-730 [8]
Li-TFSI + *t*BP	25.86%*	25.65%*	*Nature* **623**, 531-537 [9]
Li-TFSI + *t*BP	26.21%*	25.89%*	*Science* **387**, 186-192 [10]
Li-TFSI + *t*BP	26.10%*	26.00%*	*Nat. Commun.* **15**, 8620-8631 [11]
Li-TFSI + *t*BP	26.64%*	25.94%*	*Nature* **635**, 82-88 [12]
Li-TFSI + *t*BP	26.28%	25.82%*	*Joule* **9**, 101879 [13]
Li-TFSI + *t*BP	26.31%*	25.55%*	*Nat. Energy* **9**, 1506-1516 [14]
Li-TFSI + *t*BP	26.10%*	25.60%*	*Nature* **633**, 359-364 [15]
Li-TFSI + *t*BP	26.17%*	-	*Nat. Commun.* **15**, 10908-10919 [16]
Li-TFSI + *t*BP	26.13*	-	*Nat. Energy* **10**, 774-784 [17]
PVK+PTAA	25.86%*	25.70%*	*Nat. Commun.* **16**, 9345-9383 [18]
**TPFB**	**26.35%***	**26.03%***	**This work**

*Certified PCE and certified quasi-steady PCE. -: No relevant characterization in the article.

References

1. Liang, C. et al. Intrinsic phase stability and inherent bandgap of formamidinium lead triiodide perovskite single crystals. *Angew. Chem.* **61**, e202212700 (2022).

2. Caprioglio, P. et al. On the origin of the ideality factor in perovskite solar cells. *Adv. Energy. Mater.* **10**, 2000502 (2020).

3. Le Corre, V, M. et al. Revealing charge carrier mobility and defect densities in metal halide perovskites via space-charge-limited current measurements. *ACS. Energy. Lett.* **6**, 1087-1094 (2021).

4. Green, M, A., Ho-Baillie, A., Snaith, H, J. The emergence of perovskite solar cells. *Nat. Photonics.* **8**, 506-514 (2014).

5. Liang, Y. et al. Toward stabilization of formamidinium lead iodide perovskites by defect control and composition engineering. *Nat. Commun.* **15**, 1707 (2024).

6. Jeon, N, J. et al. A fluorene-terminated hole-transporting material for highly efficient and stable perovskite solar cells. *Nat. Energy.* **3**, 682-689 (2018).

7. Ren, Y. et al. A spiro-OMeTAD based semiconductor composite with over 100°C glass transition temperature for durable perovskite solar cells. *Nano. Energy.* **81**, 105655 (2021).

8. Park, J. et al. Controlled growth of perovskite layers with volatile alkylammonium chlorides. *Nature*. **616**, 724–730 (2023).

9. Huang, Z. et al. Anion–π interactions suppress phase impurities in $FAPbI_3$ solar cells. *Nature*, **623**, 531-537 (2023).

10. Zai, H. et al. Wafer-scale monolayer MoS_2 film integration for stable, efficient perovskite solar cells. *Science*. **387**, 186-192 (2025).

11. Qu, Z. et al. Enhanced charge carrier transport and defects mitigation of passivation layer for efficient perovskite solar cells. *Nat. Commun.* **15**, 8620-8631 (2024).

12. Li, S. et al. High-efficiency and thermally stable $FACsPbI_3$ perovskite photovoltaics. *Nature.* **635**, 82-88 (2024).

13. Kim, J. et al. Susceptible organic cations enable stable and efficient perovskite solar cells. *Joule.* **9**, 101879 (2025).

14. Li, Q. et al. Harmonizing the bilateral bond strength of the interfacial molecule in perovskite solar cells. *Nat.*

Energy. **9**, 1506-2516 (2024).

15. Liu, C. et al. Two-dimensional perovskitoids enhance stability in perovskite solar cells. *Nature.* **633**, 359-364 (2024).

16. Shen, L. et al. Ultrathin polymer membrane for improved hole extraction and ion blocking in perovskite solar cells. *Nat. Commun.* **15**, 10908-10919 (2025).

17. Seo, G. et al. Efficient and luminescent perovskite solar cells using defect-suppressed SnO_2 via excess ligand strategy. *Nat. Energy* **10**, 774-784 (2025).

18. Tang, H. et al. Confining iodide migration with quantified barrier for durable perovskite solar cells. *Nat. Commun.* **16**, 9345-9383 (2025).
